# Sustainably cultured coral scaffold supports human bone marrow mesenchymal stromal cell osteogenesis

**DOI:** 10.1016/j.reth.2024.06.002

**Published:** 2024-06-29

**Authors:** Chiara Gentili, Maria Elisabetta Federica Palamà, Gillian Sexton, Sophie Maybury, Megan Shanahan, Yeyetunde Yvonne Omowunmi-Kayode, James Martin, Martin Johnson, Kerry Thompson, Owen Clarkin, Cynthia M. Coleman

**Affiliations:** a Department of Experimental Medicine (DIMES), University of Genova, Genova, Italy; b College of Medicine, Nursing and Health Science, School of Medicine, Regenerative Medicine Institute (REMEDI), University of Galway, Galway, Ireland; c DCU Biomaterials Research Group, Centre for Medical Engineering Research, School of Mechanical and Manufacturing Engineering, Dublin City University, Dublin 9, Ireland; d Zoan Nuáil Teoranta T/A Zoan BioMed, The Hatchery Building, Cloonacarton, Recess, Galway, Ireland; e Ecodiversity Ltd, Derryconnell, Schull, Co. Cork, Ireland; f College of Medicine, Nursing and Health Science, School of Medicine, Anatomy Imaging and Microscopy Facility, University of Galway, Galway, Ireland

**Keywords:** Coral scaffold, Bone grafting substitute, Mesenchymal stromal cell, Mesenchymal stem cell, Osteogenesis, Bone

## Abstract

The current gold standard grafting material is autologous bone due to its osteoinductive and osteoconductive properties. Autograft harvesting results in donors site morbidity. Coral scaffolds offer a natural autograft alternative, sharing the density and porosity of human bone. This study investigated the biocompatibility and osteogenic potential of a novel, sustainably grown *Pocillopora* scaffold with human bone marrow-derived mesenchymal stromal cells (MSCs). The coral-derived scaffold displays a highly textured topography, with concavities of uniform size and a high calcium carbonate content. Large scaffold samples exhibit compressive and diametral tensile strengths in the range of trabecular bone, with strengths likely increasing for smaller particulate samples. Following the *in vitro* seeding of MSCs adjacent to the scaffold, the MSCs remained viable, continued proliferating and metabolising, demonstrating biocompatibility. The seeded MSCs densely covered the coral scaffold with organized, aligned cultures with a fibroblastic morphology. *In vivo* coral scaffolds with MSCs supported earlier bone and blood vessel formation as compared to control constructs containing TCP-HA and MSCs. This work characterized a novel, sustainably grown coral scaffold that was biocompatible with MSCs and supports their *in vivo* osteogenic differentiation, advancing the current repertoire of biomaterials for bone grafting.

## 1 Introduction

Bone has the capacity to repair and remodel [1]. However, bone grafting may be required following orthopaedic trauma, oncological surgery, revision arthroplasties, infection or upon delayed/non-union fracture healing. Comorbidities such as cancer [2] and diabetes [3] may also require bone grafting to support fracture repair. The optimal bone graft is biocompatible, has a degradation profile balanced with the rate of osteoid deposition, contains pores with a size and density to allow cell infiltration, angiogenesis and nutrient transfer, and has mechanical properties to bear weight [4]. Although synthetic bone graft substitutes, such as Vitoss [5], are available on the market, autografts represent the majority of bone grafts [6,7] as they are histocompatible, eliminate the risk of disease transfer, and are both osteoconductive and osteoinductive. However, their harvest is associated with chronic pain, nerve injury, pelvic instability, arterial injury, tumour transplantation, large loss of blood and extended anaesthesia time [[8], [9], [10], [11]].

Investigations into the efficacy of coral scaffolds to support bone growth have been ongoing for decades [[12], [13], [14]] given its similarity in chemical composition and porosity to that of bone [15]. Each species of coral naturally offers distinct pore sizes with varying levels of interconnectivity, thereby providing a bespoke capacity support blood vessel ingrowth, nutrient exchange, cell attachment/proliferation, weight bearing capacity and degradation profile to compliment the intended application [16]. A variety of coral genera, such as *Porites* [[17], [18], [19]], *Goniopora* [16], *Madrepora* [20], *Pocillopora* [21,22], *Montipora* [23] and *Acropora* [24] have been characterized for their physical and osteogenic properties, resulting in the launch of wild-harvested clinically approved grafting products such as Biocoral, Pro-osteon and Interpore [25]. Published reports comparing the resorption rates of naturally occurring *Porites, Goniopora, Favites, Lobophyllia* and *Acropora* scaffolds in both orthotopic and ectopic sites concluded that the resorption rate was dictated by the scaffold's porosity volume, calcium density and grafting location [13,26]. Some groups described the rapid degradation of *Porites* granules [[27], [28], [29]] therefore supporting *Acropora* scaffolds for clinical application, while other investigators concluded there was potential for improvement on *Acropora* scaffolds given the number of non-unions remaining at the conclusion of their study [30]. Contrary reports demonstrated the slow resorption rate of *Porites* in human iliac crest applications, resulting in a defect remaining at the completion of the study time course [31].

In an attempt to enhance their efficacy, coral scaffolds have been combined with platelet rich plasma [32], collagen scaffolds [33], osteogenic recombinant proteins [34,35], angiogenic proteins [36,37], proliferative proteins [38], osteoblasts [39], adipose-derived mesenchymal stromal cells (MSCs) [40] and bone marrow-derived MSCs [41]. *In vivo* investigations combining *Acropora* or *Porites* with cells reported a scaffold resorption profile balanced with osteoid deposition, an improvement over the coral scaffold alone [42,43]. However, the literature remains conflicted regarding the efficacy of a coral scaffold in comparison to autograft [41], an expected outcome given the wide variety of *in vivo* models employed, constructs investigated and bespoke nature of these therapies.

Coral scaffolds hold great promise as a bone graft substitute or expander. However, its clinical success depends on identifying the optimal combination of coral species with cellular compatibility, resorption profile and mechanical strength tailored for the clinical application. To expand the repertoire of scaffolds available for orthopaedic repair, this study investigated the biocompatibility and osteogenic potential of sustainably grown *Pocillopora* granules when combined with adult human bone marrow derived MSCs. Zoan BioMed, the producer of the *Pocillopora* granules used herein, has patented technology enabling the sustainable, rapid, clonal culture of tropical coral, followed by their processing the resultant aragonite skeleton into medical device-grade materials for bone grafting applications as per ISO13485:2016. This technology advances the current state of the art by avoiding wild-harvested coral and offering a wider variety of species beyond the traditional *Acropora* and *Porites*. Furthermore, this approach preserves the coral reef ecosystem from the over-collection of the wild coral avoiding the destruction of reef habitat and reduced biodiversity.

## 2 Methods

### 2.1 Coral scaffold preparation

Coral scaffolds (Zoan Biomed) were produced from coral grown under quality management system-controlled conditions in a bespoke aquaculture facility, fully detailed in Ref. [44]. Briefly, coral was harvested from culture and cut into pieces of 20 mm ± 5 mm length. To remove organic matter, the coral pieces were incubated in 5% (w/v) sodium hypochlorite for 5 h and subsequently rinsed 4–5 times in prepared reverse osmosis/deionized (RO/DI) water, followed by an RO/DI water soak for 30 ± 5 h The coral was dried at 90 °C and evaporated loss was measured regularly until there was no more than a ± 0.2 g difference in the weight between timed intervals of the material.

Scaffolds used for *in vivo* evaluation were then reduced to a 1–2 mm specification using a benchtop disk mill. The outputted material fractions were sieved using standardized aperture sized wire mesh sieves as per ISO 3310/ASTM E11 to attain the correctly fractioned material. The particle size distribution of 1–2 mm is confirmed using a validated method as described under DIN 66165-2:2016-08 where 85 to 95 wt.% of the coral particles have a particle size between 1.0 and 2.0 mm.

### 2.2 Chemical scaffold characterisation

Energy-dispersive X-ray (EDX) analysis was conducted on a Hitachi S-4700 SEM with EDX Analysis system (Hitachi, Krefeld, Germany). Using INCA software (ETAS Ltd, Derby, UK), a flat area of interest was identified on the external surface of each sample at x150 magnification. EDX reproducibility (and therefore precision) of quantitative elemental composition is affected by non-smooth surfaces such as those found in biominerals. Quantification of carbon (C), oxygen (O) and calcium (Ca) was performed in technical triplicate or quadruplicate. For each element of interest on each sample type, repeat measurements were used to derive mean values and 95% confidence interval on the mean values were generated by applying the student's T distribution for small sample sizes.

Fourier Transform Infrared Spectroscopy (FTIR) for the characterisation of chemical structure of the coral scaffolds was conducted using a Shimadzu FTIR spectrophotometer-8300 connected to an AIM-8800 microscope (Shimadzu, Duisburg, Germany). An FTIR spectrum of calcium carbonate in the calcite form (Sigma–Aldrich, Wicklow, Ireland) served as a reference material. Measurements were taken in transmittance (T%) mode under Happ-genzel apodization. Each sample was scanned 20 times at a resolution of 4 cm^−1^ in the wavelength range of 500–4000 nm. A background sample of air was taken before use and in between the measurement of each sample.

### 2.3 Determination of compressive strength

Compression Strength (CS) testing was carried out by adhering to ISO9917:2007 as closely as possible (n = 5). All compression samples were cut using wire cutters and abraded at either end using silica carbide paper to produce samples of 4 mm diameter and 6 mm height. Where individual branches of coral did not conform correctly to the standard a diameter:height ratio of 1:1.5 was always maintained, with an error range of ±0.2 mm. The coral samples were compressed using a mechanical testing machine (Z005, Zwick/Roell, Germany) equipped with a 5 kN load cell at room temperature. A 0.005 N pre-load was applied. The samples were compressed to fracture at a crosshead speed of 1 mm/min. Peak load was used for calculation of compressive strength.

(1) $$\sigma_{c}=\frac{P_{cf}}{A}$$

where σ_c_ is the Compressive Strength (Pa), P_cf_ is the load at fracture (N) and A is the Cross-sectional Area of the sample (m).

### 2.4 Determination of fracture toughness

Following the ISO6872:2015 standard, coral samples (n = 5) are cut from the coral core using a Diamond Precision Cutter and abraded down using silicon carbide paper to a beam shape on a grinding and polishing machine. Each beam had length of 16–25 mm, width of 4 mm and thickness of 4 mm. A notch was cut into the centre of the beam length of each sample using a razor blade to create a single-edge notched beam (SENB). The stress intensity shape factor, γ, was calculated for each sample using the following formula:

(2) $$\gamma =1.9109-5.1552\alpha \mp 12.68802\alpha^{2}-19.5736\alpha^{3}+15.9377\alpha^{4}-5.1454\alpha^{5}$$

where α is the relative depth, calculated by a/w, where a is the average notch depth and w is the thickness of the beam. This equation applies to testing where S_1_/w = 5 only (see below). Samples were placed on a mechanical testing machine (Z005, Zwick/Roell, Germany) fitted with a 200 N load cell. Samples were loaded to open the crack from the notch at a loading rate of 1 mm/min. The equation used to calculate the fracture toughness of each specimen is as follows.

(3) $$K_{IC}=\frac{F}{b\sqrt{w}}\times \frac{S_{1}-S_{2}}{w}\times \frac{3\sqrt{\alpha }}{2{\left(1-\alpha \right)}^{1.5}}\times \gamma$$

where K_IC_ is the fracture toughness (MPa.m^1/2^), F is the fracture load (MN), b is the beam width (m), w is the beam thickness (m), S_1_ is the support span (m), γ is the stress intensity shape factor and S_2_ is equal to 0 (true for three-point bend testing).

### 2.5 Determination of diametral tensile strength

Diametral Tensile Strength (DTS) was carried out according to Chow et al. [45], where n = 30. All DTS samples were cut using wire cutters and abraded at either end using silica carbide paper to produce samples of 6 mm diameter and 3 mm width. Where individual branches of coral did not conform correctly to the standard a diameter:width ratio of 2:1 was always maintained, with an error range of ±0.2 mm. For diametral tensile testing compressive load was applied in the diametral plane of a cylindrical specimen, perpendicular to the longitudinal axis (Fig. 2A). The coral samples were compressed using a mechanical testing machine (Z005, Zwick/Roell, Germany) equipped with a 5 kN load cell. A 0.005 N pre-load was applied. The samples were compressed to fracture at a crosshead speed of 1 mm/min. Peak load was used for calculation of compressive strength.

(4) $$\sigma_{DT}=\frac{2P_{t}f}{\pi dt}$$

where $\sigma_{DT}$ is the Diametral Tensile Strength (Pa), $P_{tf}$ is the Load at fracture (N), d is the diameter (m) and t is the thickness (m) [46].

### 2.6 Determination of Weibull modulus and characteristic strength

The Weibull modulus was obtained by plotting a Weibull distribution curve from the experimental results as per ISO20501:2019. This was performed by gathering stress data and ranking it from lowest to highest. From this, values could be plotted, and Weibull modulus and characteristic strength attained. The Weibull probability function is as follows:

(5) $$P_{f}\left(\sigma \right)=1-\exp\left\{-{\left(\frac{\sigma_{t}f}{\sigma_{c}}\right)}^{m}\right\}$$

where $P_{f}\left(\sigma \right)$ is the probability of failure, $\sigma_{tf}$ is stress at failure, σ_c_ is the characteristic failure strength and m is the Weibull modulus.

### 2.7 Modelling the Effect of Notch and Crack Size on Strength

The effect of notch and crack size on the strength of the coral material was modelled using a Linear Elastic Fracture Mechanics (LEFM), Theory of Critical Distances (TCD) and a TCD combined with a Weibull Stress Reduction Factor approach. This allows us to consider the effect of porosity reductions on strength of the coral scaffolds as we reduce the particle size through milling and infer strengths on smaller samples that are not easily mechanically tested.

TCD is a group of methods, outlined by Taylor et al. [47], used to predict the effect of notches (or pores) on failure stresses using a characteristic length parameter, L, the critical distance, which can be calculated using the following equation:

(6) $$L=\frac{1}{\pi }{\left(\frac{K_{C}}{\sigma_{0}}\right)}^{2}$$

where K_C_ is the fracture toughness (in MPa.m^1/2^) and σ_0_ is the strength of the material containing no defects. Using L, the following equation was used to predict the tensile failure stress, $\sigma_{tf}$.

(7) $$\sigma_{tf}=\frac{\sigma_{0}}{\left[1+\frac{a}{2L}\left(1-\frac{1}{{\left(1+\frac{2L}{a}\right)}^{3}}\right)\right]}$$

where, a is the length of a given notch. The σ_0_ value used was 16.99 MPa, which was the highest Diametral Tensile Strength calculated empirically. Analysis was carried out for sharp cracks (using a Linear Elastic Fracture Mechanics approach) and holes (using the TCD and TCD with a Weibull Stress Reduction Factor approaches). This replicates approaches carried out by Taylor et al. [48]. For LEFM approach, the following equation was used, where Y is a geometric factor equal to 1:

(8) $$\sigma_{tf}=\frac{K_{C}}{\left(Y\sqrt{\pi a}\right)}$$

### 2.8 Bone marrow MSC isolation and culture for biocompatibility assessment

Bone marrow-derived human MSCs were isolated from purchased bone marrow (Lonza) or isolated from the iliac crest bone marrow of informed, consenting adult human donors from the Galway University Hospital Group under institutional approval CA 02/08 using the methods previously described [49]. The MSCs were seeded at a density of 5700 cells/cm^2^ with expansion media consisting of alpha-MEM Medium GlutaMAX (Gibco) supplemented with 10% fetal bovine serum (FBS; HyClone ThermoFisher), 1% penicillin and streptomycin (Gibco) and recombinant human fibroblast growth factor (Peprotech) to a final concentration of 1 ng/mL (for proliferation, confocal microscopy and osteogenesis) or 5 ng/ml (for viability, metabolism, necrosis). The cultures were maintained in a humidified incubator at 37 °C and 5% CO_2_. The protocols employed in the current study have been validated with 100+ donors to isolate MSCs that are CD90^+^, CD73^+^ and CD105^+^ with osteogenic differentiation potential [49].

### 2.9 Cell viability

To conduct *in vitro* biocompatibility assays, coral segments were fragmented with an arbour press and separated by particle size using a metal sieve with 0.01 cm^2^ opening diameter. Smaller particles were discarded with larger particles being retained on the sieve. Individual pieces of coral were measured with a micrometer and their volume and surface area calculated to ensure A) uniform dosing of coral scaffolds to cells in monolayer during assays and B) to determine cell plating density.

MSCs from three donors were seeded in technical duplicate at a density of 5200 cells/cm^2^ onto a 96-well plate in MSC expansion medium and incubated at 37 °C and 5% CO_2_. After allowing cells to adhere for 24 h, coral was placed in the wells to cover 10% of the well surface area as per ISO10993-5:2009. Positive control wells contained MSCs cultured in a monolayer without coral. The negative control samples were MSCs without coral, but with the induction of necrosis by the addition of 2% TritonX-100 (Sigma–Aldrich). At data collection, the coral scaffolds were removed from each well and the viability of the cells within the well assayed with the LIVE/DEAD Cytotoxicity Kit for mammalian cells (Thermofisher) according to the manufacturer's instructions. To visualize the cells, an inverted fluorescent microscope (Olympus IX71) was used with CellSens software using GFP and TRITC filters at an exposure of 580 ms, ISO 1600 and a resolution of 1360x1024.

### 2.10 Cell proliferation

To determine the proliferative capacity of the MSCs when cultured adjacent to the devitalised coral scaffold, the culture's DNA content was quantified using a Quant-iT PicoGreen dsDNA Assay Kit (ThermoFisher) according to the manufacturer's instructions. MSCs from 3 donors in technical triplicate were seeded onto a 96-well plate at a density of 5200 cells/cm^2^ and incubated at 37 °C and 5% CO_2_ in a humidified incubator. Day 0 samples were harvested 3 h after plating. After allowing the cells to adhere for 24 h, the coral scaffold was placed in the wells to cover 10% of the well surface. Positive controls consisted of cells seeded onto the plates without the coral scaffolds and negative controls were cells killed by induction of necrosis with 4% TritonX-100 for 20 min prior to harvest. DNA content was quantified daily over 4 days of culture with the Quant-iT kit using a Thermo Scientific Varioskan Flash Spectral Scanning Multimode Reader and SkanIt Software 2.4.3 RE for Varioskan Flash. The fluorescence of each sample was measured at 535 nm. To determine the absolute number of cells in each well, the DNA content was divided by 6 pg [50]. The cell number was normalised to day 0 values.

### 2.11 Metabolic analysis

To measure the metabolic activity of cells when seeded adjacent to coral scaffold, a CellTiter 96 Aqueous Non-Radioactive Cell Proliferation Assay (MTS) kit was utilised as per the manufacturer's instructions. With 3 biologic donors plated in technical duplicate, the MSC plating density and culture conditions mirrored those described in the proliferation assay. The conditioned medium was collected after 3 days of co-culture and assayed at 490 nm (0.1 s) using a 1420 Multilabel Counter (VICTOR3V) and Wallac 1420 software.

### 2.12 Quantifying necrosis

To quantify cytotoxicity because of exposure to the coral scaffold, a lactose dehydrogenase (LDH) assay was performed using a Cytotoxicity Detection kit (Roche) as per the manufacturer's instructions, again using the experimental design described in the proliferation assay. The positive control was conditioned medium from MSCs cultured without coral but with the induction of necrosis with 2% TritonX-100 for 30 min. The negative control was conditioned medium from MSC cultured without coral. Conditioned medium from three MSC donors was assayed in technical duplicates at 490 nm for 0.1 s using a 1420 Multilabel Counter (VICTOR3V) and Wallac 1420 software. Following analysis of samples, the average of each sample was determined before the background absorbance level (cytotoxic reagent with medium only control) was subtracted from each reading.

### 2.13 Scanning electron microscopy

For those samples with MSCs, the cells were seeded onto the coral scaffold at a density of 6000 cells/cm^2^ at room temperature for 3 h in rotation. The cell-scaffold construct was cultured in static conditions for 14 days in a humidified incubator at 37 °C in 5% CO_2_. The MSCs were maintained in cell expansion media with twice weekly media changes. They were then fixed in 2% glutaraldehyde (Sigma–Aldrich) and 2% paraformaldehyde (PFA) in 0.1 M sodium cacodylate/HCl buffer at a pH of 7.2.

All samples were washed in 0.1 M sodium cacodylate/HCl buffer and exposed to iodine vapour overnight. The MSC-scaffold constructs were then dehydrated through a series of gradedethanols (Sigma–Aldrich) and incubated in HMDS (Sigma–Aldrich) at room temperature for 20 min to remove any remaining moisture, then air dried overnight. The samples were affixed onto a carbon stub and coated with an ultrathin layer of gold under low vacuum using an EMscope SC500 (Bio-Rad) sputter coater. Images were collected on a Hitachi S-2600 N variable pressure SEM.

### 2.14 Confocal microscopy

The MSCs were seeded onto coral scaffold as described for SEM imaging. To perform confocal microscopy, the MSC-scaffold constructs were fixed with 4% PFA for 15 min, washed in DPBS and permeabilized using 0.1 % TritonX-100 for 2 min at room temperature. The samples were blocked in 3% normal goat serum for 15 min, washed and stained with Hoechst 33342 (ThermoFisher Scientific) diluted 1:1000 in DPBS. Actin microfilaments were stained with Alexa Fluor 488 nm phalloidin (ThermoFisher Scientific) diluted 1:200 in DPBS in darkness at room temperature for 20 min. The samples were washed in DPBS and transferred to an IBIDI glass bottom 35 mm dish containing DPBS for imaging. Z stacks were collected using an Andor Revolution Spinning Disc Confocal Microscope with Andor IQ software where images were acquired at roughly 4.5 μm apart through the depth of the sample. Samples were illuminated using the 405 nm and 488 nm lasers. Appropriate exposure and gain were established and recorded to the channel of illumination prior to imaging. Maximum intensity Z projections were constructed using FIJI/ImageJ2.

### 2.15 *In vivo* ectopic bone formation

All *in vivo* work was performed with ethical approval (Ministro della Salute- Autorizzazione N° 1233/2020-PR), in compliance with the EU Directive 2010/63/EU for animal experiments. Two biomaterials were used in this study: (i) granules of coral (Zoan BioMed), (ii) granules containing tricalcium phosphate (TCP) and hydroxyapatite (HA), with a HA/TCP ratio of 20/80.

The human MSCs were isolated from bone marrow aspirates derived from patients undergoing total or partial arthroscopy, after informed consent (CER Liguria: 372/2019). Briefly, bone marrow was centrifuged at 300 g for 10 min and nucleated cells were cultured complete medium containing α-MEM Glutamax medium (Gibco), supplemented with 10% FBS (Gibco), 100 U/ml penicillin/streptomycin (Euroclone) and 1 ng/ml fibroblast growth factor 2 (Prepotech). The MSCs were maintained in a humidified incubator, at 37 °C and 5% CO_2_. At 90% confluence, they were detached by using trypsin/EDTA (Euroclone) and used for further experiments. Only early passage MSCs (passage 2) were used for the experiments described below.

To evaluate the *in vivo* osteogenic capacity of both scaffolds, the ectopic bone formation assay was adopted [51]. Early passaged MSCs (P2) were trypsinized and 2 × 10^6^ cells were seeded onto either coral or TCP-HA granules (50 mg/each) and encapsulated in fibrin glue (TISSEEL, Baxter) to obtain 0.5 cm Ø constructs. CD-1 mice (CD-1 nu/nu female 6 weeks old; Charles River) were anesthetized with intraperitoneal injection of ketamine/xylazine, the surgical site was cleaned with betadine, and subcutaneous pockets were created on the dorsum. The ceramic-MSCs constructs were implanted subcutaneously in the pockets and the wound was closed with chirurgical metallic graphs (7,5 x 1,75 mm, Michel). A scaffold without MSCs was also included in each animal. Groups of 6 animals were sacrificed after 15, 30, 60 and 90 days by CO_2_ saturation and the implants harvested. All animals were maintained as required by the Italian Ministry of Health in accordance with the standards of the Federation of European Laboratory Animal Science Associations (FELASA).

### 2.16 Paraffin and resin histology

Explants were fixed in 3.7% PFA in PBS, decalcified with 0.5 M EDTA, dehydrated in ethanol, and paraffin embedded. Cross sections of 5 μm were cut on a RM2165 microtome (Leica Microsystems), dewaxed and stained according to the appropriate histological analysis: haematoxylin and eosin (H&E) staining to observe bone formation, Mallory staining to assess vessel formation and Goldner-Masson trichrome staining to distinguish deposited collagen fibres.

For resin embedding, non-decalcified, fixed samples were dehydrated and infiltrated with the light-curing resin Technovit 7200VLC (Kulzer) for 21 days with resin replaced every 7 days. Samples were polymerized by the EXAKT 520 polymerization system (EXAKT) with curing performed with 450 nm light at temperature below 40 °C. Sections were cut using the EXAKT 310 CP cutting unit. Obtained sections were approximately of 150 μm in thickness and were then ground to 20–30 μm thickness using the EXAKT 400 CS micro grinding unit. Images were acquired by AxioPhot microscope (Carl Zeiss) at different magnifications.

### 2.17 Histomorphometry analysis

To quantify deposited bone and vascular structures, 4 μm sections were stained with H&E and Mallory's trichrome, respectively. Images were acquired by using an AxioPhot microscope and Fiji ImageJ was used for quantitative measurements. To calculate bone volume, photomerges of five non-consecutive sections were generated by Photoshop. Bone and scaffold areas were measured and the ratio between both areas converted to percentage of bone per region of interest (ROI). To analyse the ability of the two biomaterials to promote new vessel formation, five non-consecutive sections for each sample were analysed. The number of vessels per ROI and the average area covered by vessels were calculated.

To assess bone formation indices, mice received two intra-peritoneal injections of calcein (10 mg/kg dissolved in 2% Na_2_HCO_3_ in 0.9% NaCl) (Sigma) 14 and 3 days before sacrifice. Calcein double labelling was quantified in resin embedded sections. Mineralizing surface (MS, um^2^) and mineral apposition rate (MAR, μm/day) were measured from unstained sections, and bone formation rate (BRF) was calculated (BFR = MS/BS ∗ MAR, μm^2^/μm^3^/day) where BS is bone surface.

### 2.18 *In vitro* angiogenesis

HUVECs (ATCC CRL-1730TM) were seeded on a T75 cm^2^ flask and cultured in HUVEC complete medium consisting of F12-K medium (ATCC), supplemented with 30 mg/ml of endothelial cell growth supplement (Sigma–Aldrich), 10% FBS, 100 U/ml of penicillin/streptomycin and 0.1 mg/ml of heparin (Pharma Tex). Passage 5 HUVECs were used for all the experiments. For each test, positive and negative controls were carried out by incubating cells in complete medium and in F12-K medium, supplemented with 100 U/ml of penicillin/streptomycin (serum-free medium, SF), respectively.

To evaluate *in vitro* angiogenesis, ceramic-MSCs scaffolds were created with MSCs from three donors. Constructs without cells were used as controls. Both cell-laden and empty constructs were cultured in 6-well plates in complete alpha MEM medium for 5 days and then washed with PBS and kept in culture again for further 2 days in serum-free medium. Conditioned medium (CM) was recovered, centrifuged at 300 g for 10 min to eliminate any dead cells and biomaterial residues and frozen at −80 °C for subsequent studies with HUVEC cells. Parallel scaffolds were fixed in 3.7% PFA for immunohistochemical analysis.

HUVECs were seeded in a 96-well plate (10,000 cells/well) and cultured in complete medium for 24 h to allow cells attachment. To synchronize cell cycle, HUVECs were incubated with SF medium for 2 h. HUVECs treatments were performed for 24 h using 200 μl of CM derived from either coral or TCP-HA ± MSCs and diluted 1:5 in SF medium. Cells proliferation was then measured with a cell proliferation ELISA (Roche) and a colorimetric BrdU proliferation assay (Roche) following the manufacturer's instruction.

To evaluate the coral and TCP-HA scaffolds’ ability to induce HUVECs migration, a transwell system (Corning) was used. Transwells were placed in a 24-well plate and 50,000 cells resuspended in SF medium were seeded inside the inserts. 200 μl of CM derived from either coral or TCP-HA ± MSCs was placed in the lower chamber. After 9 h of devitalization at 37 °C and 5% CO_2_, inserts were washed in PBS, and the non-migrated cells in the upper part of the membrane were gently removed using a cotton swab. Migrated cells were fixed in 3.7% PFA for 10 min and stained with 1% methylene blue for 30 min. Images were acquired with an inverted phase microscope (Leica DMi1) and analysis was performed on at least 5 different areas, by using ImageJ Cell counter plug in. Data were normalized to the positive control.

### 2.19 Statistical analysis

*In vitro* and *in vivo* data were statistically assessed using GraphPad Prism Versions 8.4.1 and 9, respectively, then displayed as mean ± standard deviation (SD). Statistical analysis of difference between multiple groups was performed using Two-way or One-way ANOVA, followed by Tukey's multiple comparison test. Level of significance was set at p < 0.05.

## 3 Results

### 3.1 Coral scaffold characterization

**Fig. 1:**
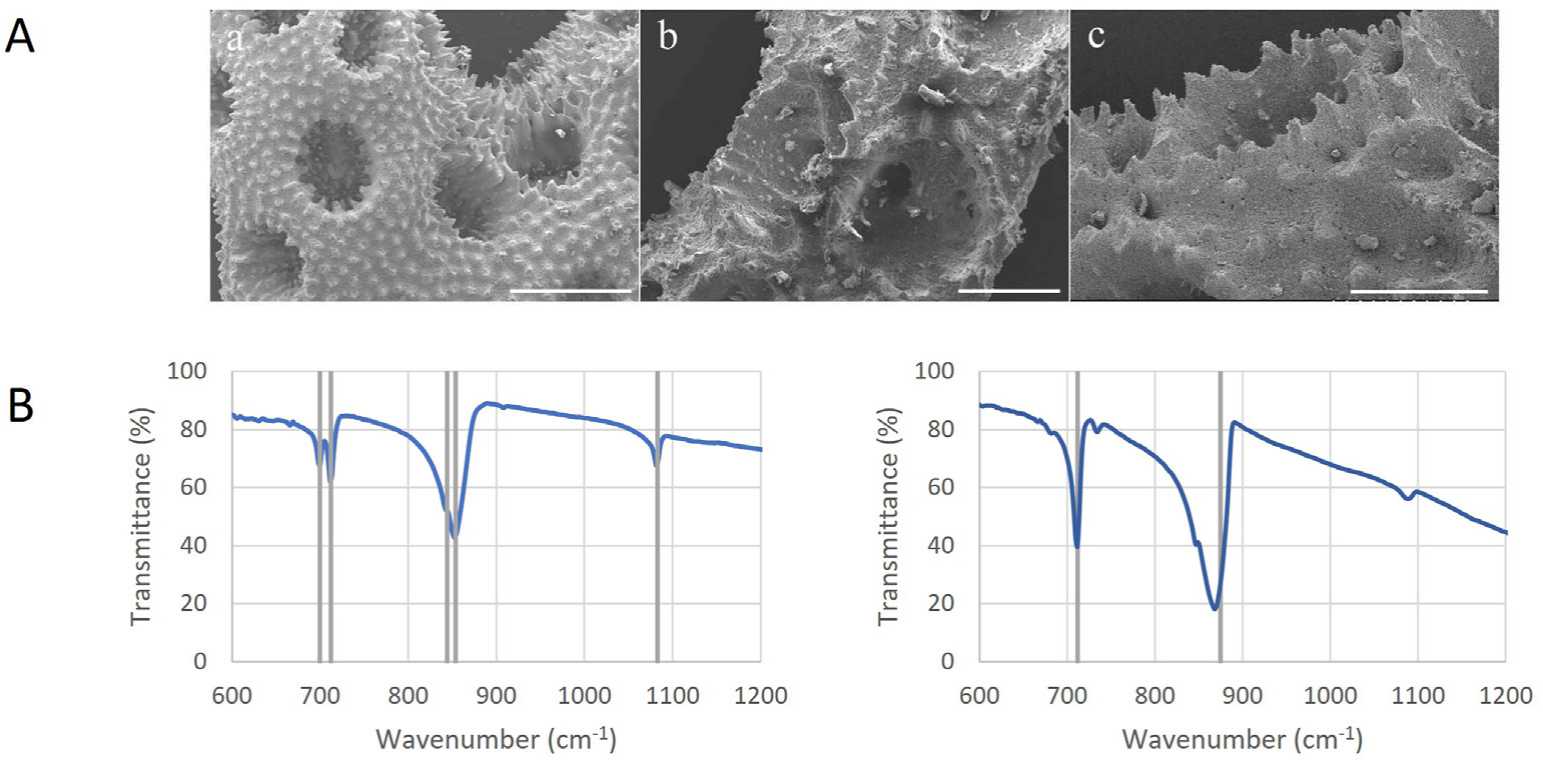
**Coral scaffold chemical characterization.** (A) SEM images of (a–c) Pocillopora scaffolds depicted the coral scaffold's highly textured, granular coenosteum with protruding verrucae. The *Pocillopora* scaffold has randomly spaced calices. The scale bars represent 1 mm in (a) and 500 μm in (b–c). (B) FTIR spectra from a *Pocillopora* scaffold (left) were aligned with that from a calcium carbonate reference sample (right). Vertical lines from the *Pocillopora* samples show the characteristic vibrational bands for aragonite while calcium carbonate standard exhibits characteristic vibrational bands for calcite [54].

**Supplemental Fig. 1:**
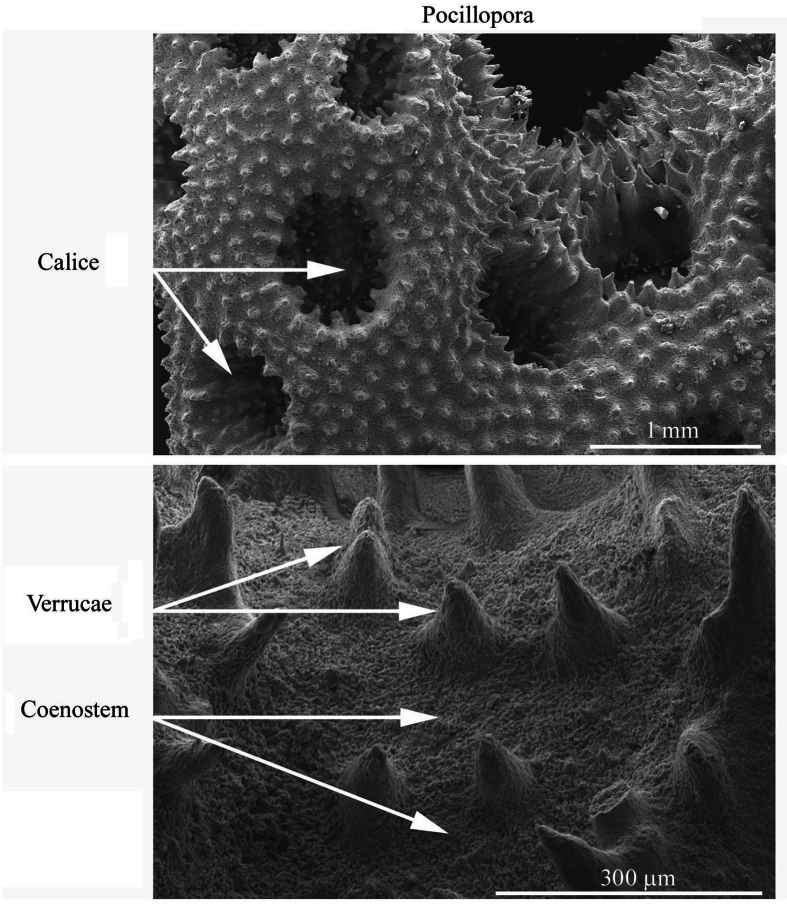
Anatomical structures of *Seriatopora* and *Pocillopora* scaffolds. SEM imaging of *Pocillopora* surface structures enabled the identification of the concave calice (top) and the flat coenosteum (bottom). The *Pocillopora* scaffold presented with superficial protuberances, or verrucae. Scale bars represent 1 mm (top) and 300 μm (bottom).

*Pocillopora* scaffolds were analysed with SEM imaging to characterize their surface topography (Fig. 1A, Supplemental Fig. 1). The anatomical structures characterized by SEM was the coenosteum, or the flat expanse of the external skeleton, the concave calices and the spine-like verrucae. The coenosteum was highly granular, complimented by the presence of spine-like verrucae with uniformly sized concave calice macro-pores that were irregularly spaced along the scaffold.

It is well established that stony corals’ skeletons are composed almost entirely of calcium carbonate, which almost without exception is in the aragonite mineral form [52,53]. EDX analysis confirms the presence of Ca, C and O in weight percent consistent with pure CaCO_3_ (Table 1). FTIR analysis was conducted on *Pocillopora* scaffolds to characterize the chemical composition and functional groups. Aragonite is known to produce vibrational bands at 1083 (v1) and 854 (v2) cm^−1^ and a characteristic double (v4) band at 700 and 712 cm^−1^ whereas calcite has a single v4 band at 71, v2 at ∼875 and no v1 band in IR [54]. Fig. 1B clearly shows strong aragonite bands as compared to Chakrabarty and Mahapatra (1999) and the calcite standard analysed during this study. Separately, evaluation of *Pocillopora* scaffolds for chemical composition and structure using ICP-MS and XRD, respectively, and found >98% calcium carbonate in the form of aragonite (data not shown).

**Table 1:** EDX chemical composition analysis. Stated ranges are 95% confidence intervals on the mean based on repeat measurements calculated using the student's T distribution. Expected results for pure calcium carbonate are shown in the final row.

	Ca (weight %)	C (weight %)	O (weight %)
*Pocillopra (n* = *4)*	36 ± 8.4	15 ± 3.3	49 ± 6.4
Pure CaCO_3_	40	12	48

### 3.2 Coral scaffold mechanical characterization

**Fig. 2:**
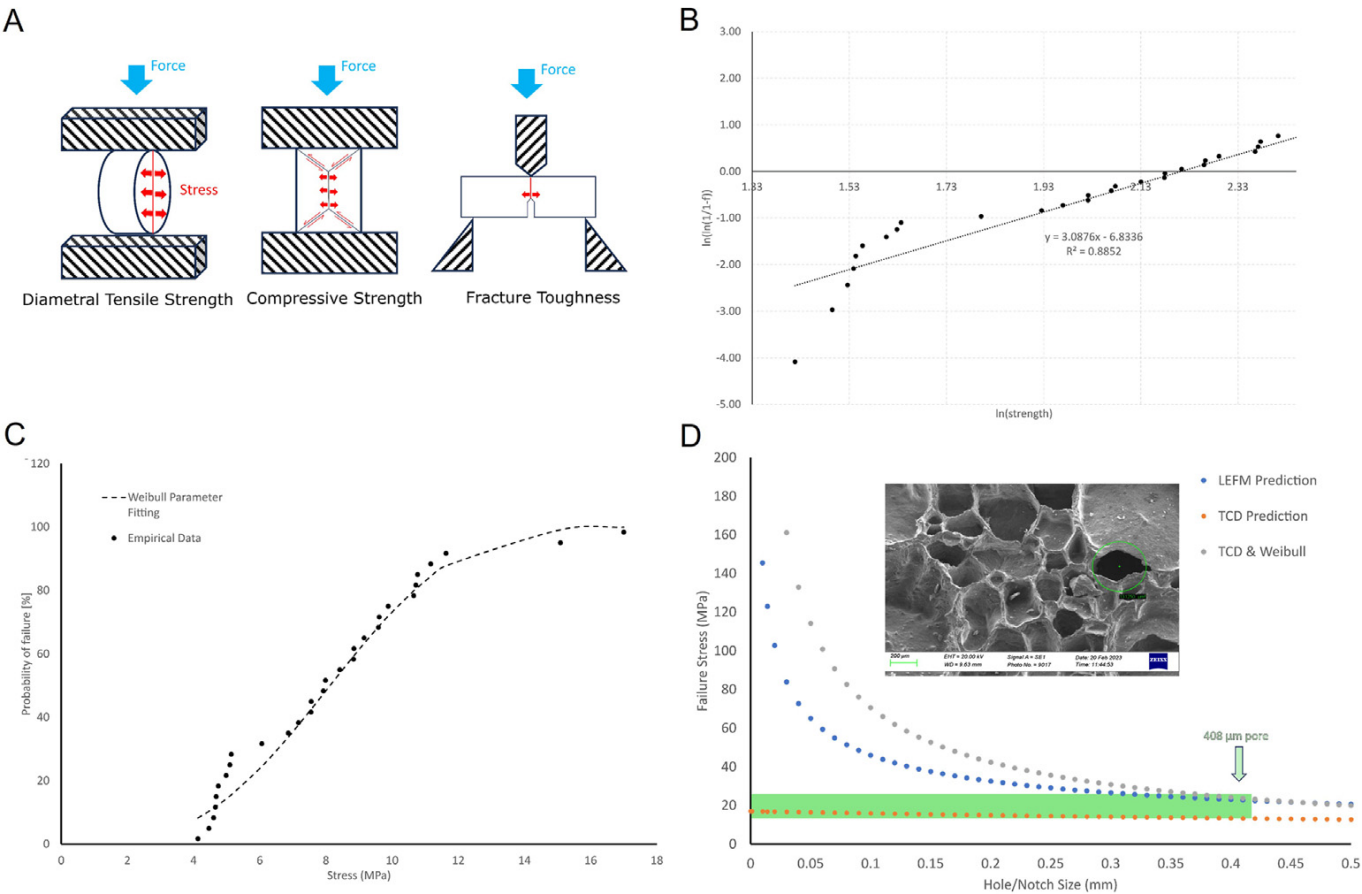
***Pocillopora* scaffold mechanical characterisation.** (A) Schematic of mechanical testing regimes. (B) Weibull distribution of diametral tensile strength data. (C) Probability of Failure by diametral tensile strength data. (D) Modelling the Effect of Notch and Crack Size on Strength using TCD, TCDW and LEFM predictions, inset image of SEM micrograph of typical largest pore size detected.

Mechanical analyses, including compressive strength, diametral tensile strength (DTS) and fracture toughness analyses were conducted on *Pocillopora* scaffolds to elucidate the mechanical integrity of the scaffolds. DTS results (n = 30) were then analysed using Weibull's probability distribution methods to indicate the probability of failure under various stressing conditions (Fig. 2B and C). As can be seen from Table 2 mean compressive strengths and DTS of 21.98 MPa and 8.02 MPa, respectively, were achieved. These results are comparable to strengths reported for several calcium phosphate cement formulations [55]. Mechanical properties varied considerably across samples, as would be expected from a naturally derived ceramic. This is further indicated by the Weibull's Modulus value of 3.09 and Characteristic Strength of 9.15 MPa. However, these results indicate greater strength and reliability to those reported for coral particles by Ma et al. (2019) [56]. Mode, I fracture toughness of the coral revealed a value of 0.815 MPa√m, indicating a relatively tough natural ceramic material.

**Table 2:** Mechanical properties of *Pocillopora* scaffolds.

	Compressive strength (MPa)	Diametral tensile strength (MPa)	Weibull's modulus	Characteristic strength (MPa)	Fracture toughness (MPa.√m)
Mean:	21.98 (n = 5)	8.02 (n = 30)	3.09 (n = 30)	9.15 (n = 30)	0.815 (n = 5)
Standard deviation:	9.41	3.60	–	–	0.306

Probability of failure vs stress is indicated in Fig. 2C, which shows that the coral material generally conforms with a Weibull distribution, though the lower strength samples do deviate from this relationship, which may be a result of the uneven natural topography and dislocations within the natural ceramic material. Modelling the effect of notch and crack size on strength (Fig. 2D) indicates that for a maximum pore size present in the ceramic material of 408 μm, as indicated by inset SEM micrograph, the predicted failure stress is likely to be in the range of 13–21 MPa, which correlates well with the upper range of mechanical data obtained empirically (7–17 MPa). Deviation between predicted and modelled results may be due to over estimation of the fracture toughness due to low sample numbers (n = 5). Reducing the fracture toughness to the lowest value achieved (0.50 MPa√m) bring the predictions within the empirical range (9.5–15 MPa). It should be noted that these results relate to relatively large coral arms and reducing the particle size will likely result in both a decreased maximum pore size, due to fractures occurring at the largest pores during milling, as well as a reduced probability of fracture, given the smaller volume over which the stress acts, that is to say a lower likelihood of a critical sized defect being present.

### 3.3 Coral scaffold biocompatibility

**Fig. 3:**
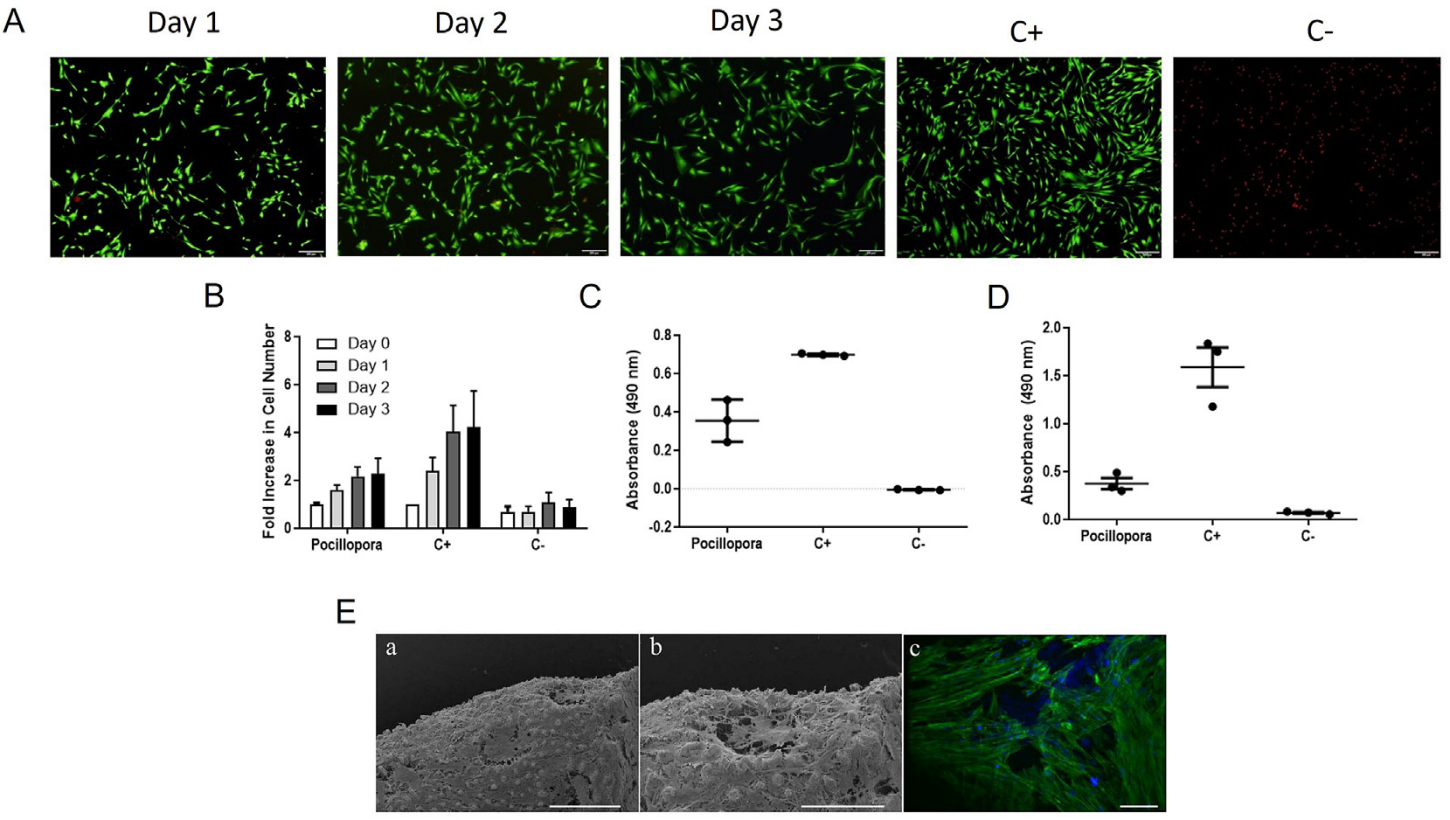
**MSCs are biocompatible with *Pocillopora* scaffolds.** (A) Live/dead fluorescent staining of MSCs through 3 days of co-culture with *Pocillopora* scaffolds. A green-stained, elongated cell body indicated healthy MSCs as indicated in the positive control (C+), while red-stained rounded structures indicated dead cells as represented in the negative control (C-). MSCs cultured adjacent to the coral scaffolds maintained their viability. The scale bars represent 200 μm. (B) MSC proliferation was evaluated over 3 days, presented as a fold increase in cell number exhibited for MSCs co-cultured with a scaffold and in controls (C+, C-) cultured in monolayer. A regular increase in cell number was observed over time in the positive control and in MSCs co-cultured with the coral scaffold. (C) The metabolic activity of MSCs co-cultured with coral scaffolds for 3 days was assayed and compared to monolayer controls. The MSCs cultured with *Pocillopora* scaffolds remained metabolically active at comparable levels to one another, but lower than that of the positive control. (D) MSC necrosis was evaluated with an LDH assay of the conditioned medium following 3 days of co-culture with coral scaffolds. There was minimal necrosis observed in MSCs cultured with *Pocillopora* scaffolds, a level comparable to MSCs cultured in monolayer. (E) SEM microscopy of MSCs seeded on (a–b) *Pocillopora* scaffolds revealed abundant fibroblastic MSCs across the coenosteum and calices of both scaffolds, indicating their adhesion and proliferation, and selective aversion to the spinules and verrucae. (c) Confocal microscopy images of MSCs seeded onto *Pocillopora* scaffolds revealed organized, aligned cultures of fibroblastic cells. Blue stain indicated nuclei and green the actin cytoskeleton. Scale bars represent (a) 1 mm, (b) 500 μm and (c) 100 μm.

The biocompatibility of MSCs cultured adjacent to a coral scaffold was investigated by viability staining of the cell monolayer following 3 days of culture (Fig. 3A). Fluorescent staining of the positive control, MSCs grown in a monolayer without coral, revealed green-stained cells that retained their characteristic fibroblastic morphology at high viability. Fluorescent staining of the negative control, MSC undergoing induced necrosis, revealed an abundance of rounding, red-stained cells. Imaging the MSCs cultured adjacent to the *Pocillopora* scaffolds revealed green-stained, viable, fibroblastic cells comparable to that of the positive control.

The proliferative capacity of MSCs cultured adjacent to *Pocillopora* scaffolds was investigated by quantifying DNA and using it to calculate cell number (Fig. 3B). The positive controls were MSCs grown in a monolayer without scaffolds while the negative control was killed via induction of necrosis. The positive control MSCs and MSCs exposed to the coral scaffolds showed a consistent fold increase in cell number over time. MSCs co-cultured with *Pocillopora* scaffolds had a comparable proliferative rate, resulting in a 2-fold increase in cell number from day 0 to day 3 while the monolayer positive control exhibited a 4-fold increase in cell number between days 0 and 3. To measure the metabolic and necrotic activity of MSCs following exposure to a coral scaffold, an MTS assay (Fig. 3C) and LDH assay (Fig. 3D) were performed on the conditioned medium following 3 days of culture. MSCs cultured adjacent to a *Pocillopora* scaffold maintained their metabolic activity and displayed a similar level of necrosis to MSCs in culture without a scaffold.

The adhesion, morphology and organization of MSCs grown on *Pocillopora* scaffolds were characterized by SEM and confocal microscopy (Fig. 3E). The MSCs on both scaffolds appeared as a dense layer of fibroblastic cells covering the majority of the coenosteum while avoiding the verrucae. Pseudopodia were observed extending from the MSCs, especially across the calice pores where the cells appeared to be cooperating to create a membrane suspended over the concavity (Fig. 3Ea, b). Complementing the SEM images, confocal microscopy of the MSC nuclei (blue) revealed the density of cells growing over the coenosteum and lining the calice pores while cytoskeletal staining (green) revealed the MSC's elongated, organized, aligned morphology (Fig. 3Ec, Supplementary Video 1). Large gaps in staining were observed in the confocal imagery where cells avoided adhering to the protruding verrucae.

Supplementary video related to this article can be found at https://doi.org/10.1016/j.reth.2024.06.002

The following is/are the supplementary data related to this article:

Supplemental Video 1 Confocal microscopy of a *Pocillopora* scaffold upon which human MSCs were cultured. Actin microfilaments were stained green and nuclei blue, enabling visualization of cell morphology and distribution on the scaffold surface. Supplemental Video 1

### 3.4 *In vivo* Neo-bone formation and maturation

**Fig. 4:**
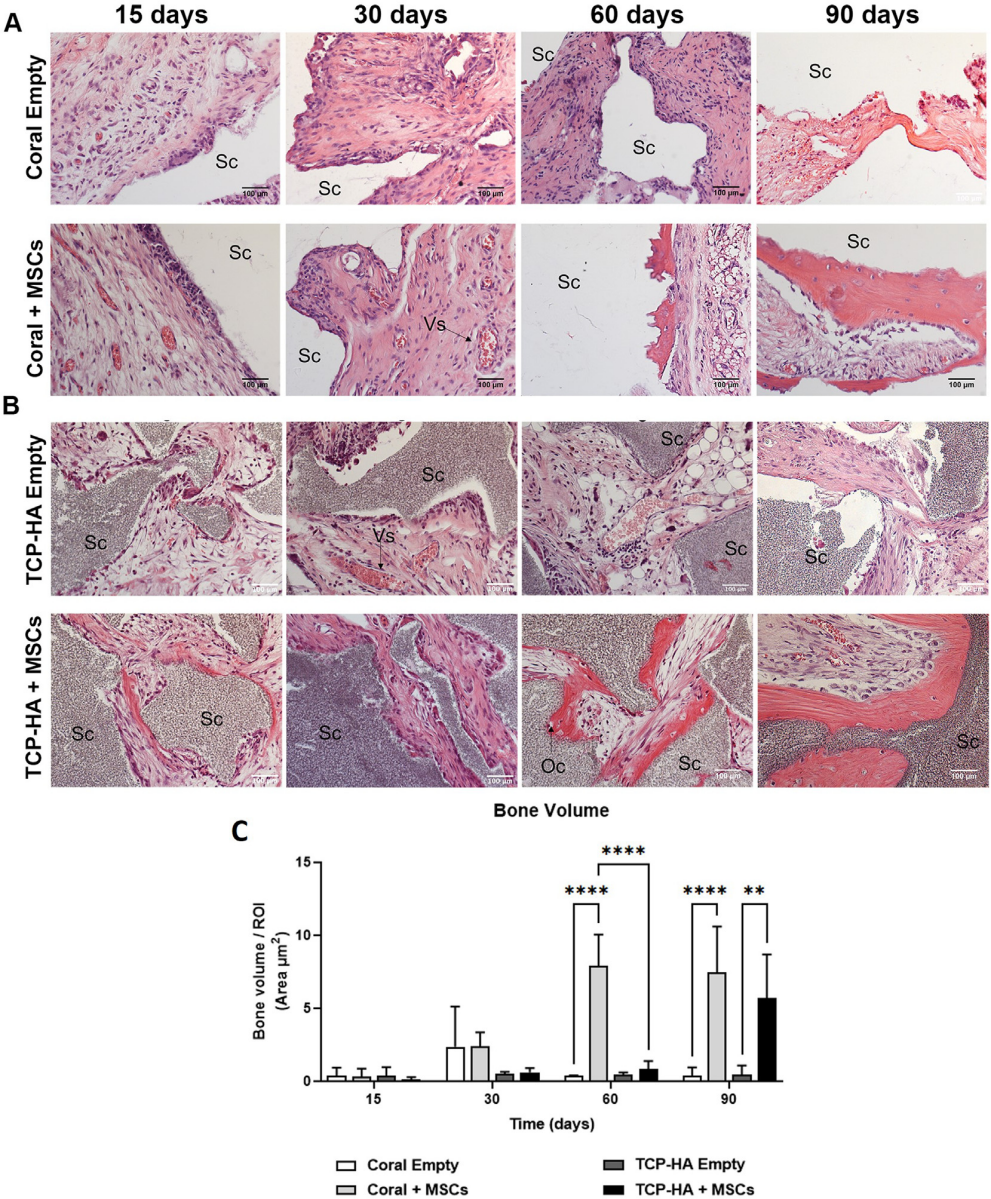
**Characterizing bone formation in H&E stained scaffolds.** The presence of scaffold (Sc), blood vessels (Vs) and osteocytes (Oc) in newly deposited osteoid (red) were observed in (A) coral and (B) TCP scaffolds with and without MSCs. Scale bars represent 100 μm. (C) Quantification of bone volume indicated a greater amount of bone at 60 days in MSC-containing coral scaffolds, then a comparable amount of bone in both MSC-containing scaffolds at 90 days. (∗∗) p < 0.01, (∗∗∗∗) p < 0.0001, two-way ANOVA.

**Supplemental Fig. 2:**
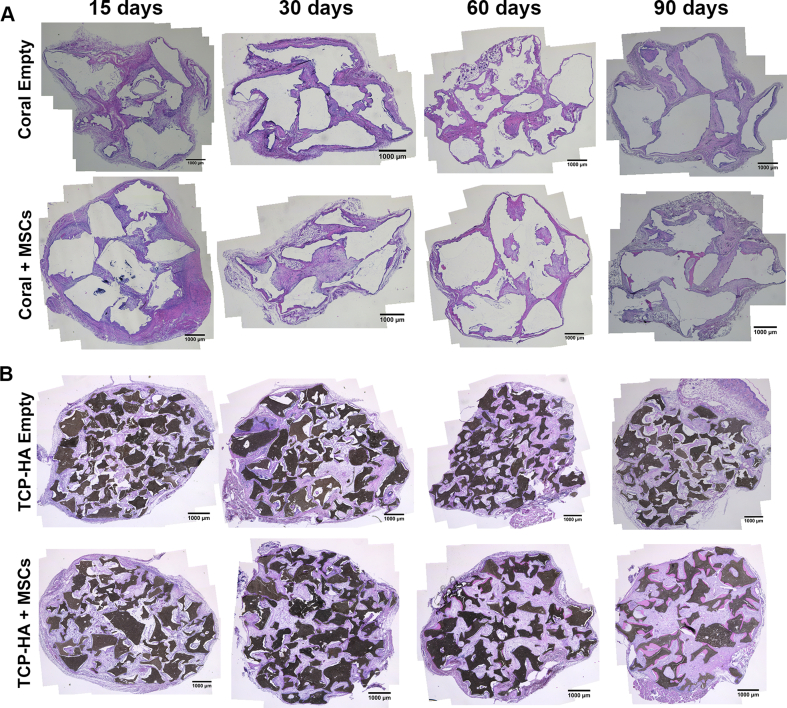
Composite images of H&E-stained scaffolds. Bone volume calculations were based on composite images of histological sections, enabling calculation of the area of bone formed by MSCs on coral (A) and TCP (B) at 15, 30, 60 and 90 days after implantation. Note the coral was dissolved in the decalcification process while the TCP remains as a dark coloured deposit.

The ability of MSCs to generate bone tissue within coral and TCP-HA scaffolds was tested in a well-established ectopic bone formation model. MSCs seeded on both coral and TCP-HA scaffolds led to new bone tissue deposition. The histological haematoxylin and eosin (H&E) staining of explants showed that the coral scaffold containing MSCs supported bone deposition after 30 days which increased in volume at 60 days and was sustained to 90 days (Fig. 4A). At 30 days it was observed that the coral implants contained immature bone rich in blood vessels (Fig. 4 arrows and Supplementary Fig. 2). More mature bony matrix was observable after 60 days, as demonstrated by progenitors with a flattened morphology lining the edge of the coral scaffold. At 90 days, mature bone matrix with embedded osteocytes was observed in coral scaffolds.

TCP-HA scaffold showed an early organization of the connective tissue in parallel fibres at 15 days (Fig. 4B). This connective tissue became denser after 30 days, when progenitor cells were found arranged in neat rows on the edges of the scaffold. After 60 days, newly formed bone tissue was observed, with osteocytes embedded in the matrix, with a considerable increase after 90 days. Both MSC-free scaffolds demonstrated no bone deposition at any time point.

Quantitative analysis enabled calculation of bone volume in each cell-scaffold construct. Photomerges of five non-consecutive sections were generated (Supplementary Fig. 2), the area of bone and scaffold was measured and the ratio between both areas converted to percentage of bone per region of interest (ROI). Histomorphometric quantification of new-formed bone indicated an increase in bone formation over time (Fig. 4C). After 60 days, coral-containing constructs contained significantly more bone volume formation compared to both its empty control and TCP-HA scaffolds, which reached a significant and measurable ossification only after 90 days.

**Supplemental Fig. 3:**
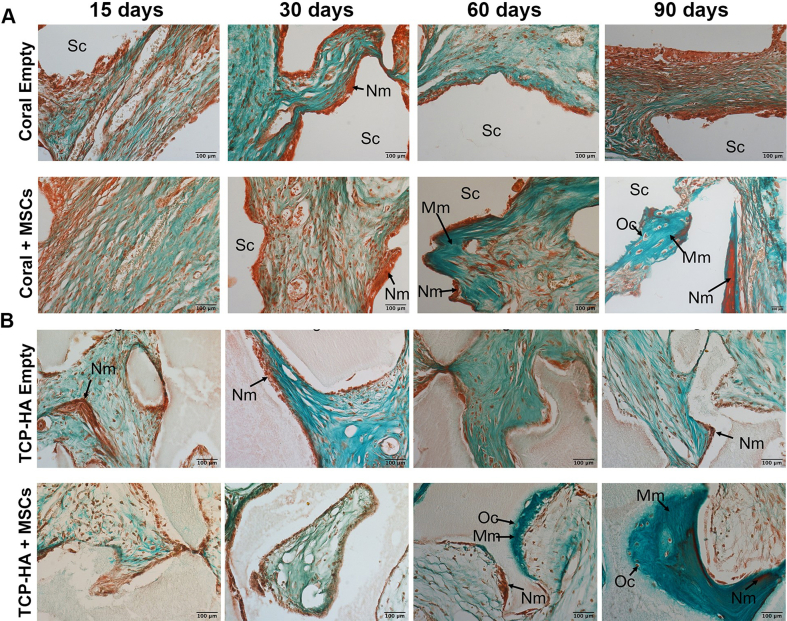
Goldner's trichrome stained scaffolds. Coral (A) and TCP (B) scaffolds with and without MSCs were explanted after 15, 30, 60 or 90 days, then stained with Goldner's trichrome to distinguish between non-calcified osteoid (brown) and calcified (green) ECM.

Matrix composition was further analysed by staining explanted scaffolds with Goldner trichrome. Osteoblasts secrete extracellular matrix (ECM) consisting primarily of type I collagen. The ECM is initially amorphous, but gradually becomes crystalline through the process of mineralization. During the latter process the collagen acts as a supporting structure and it is covered with calcium phosphate crystals. With Goldner's trichrome it was possible to distinguish the non-calcified osteoid tissue in brown, rich of collagen fibers, from the already calcified area of bone in green (Supplemental Fig. 3). Osteoid tissue was detectable in both coral and TCP-HA scaffolds after 15 days. However, the process of mineralization and mature bone matrix formation was detectable only after 60 days, confirming the physiological trend underlying osteogenesis. Interestingly, coral scaffolds showed a larger portion of newly deposed matrix (Nm) after 90 days as compared to TCP-HA scaffolds, suggesting the presence of matrix-deposition centres of active osteoblasts. It should be noted that extensive brown and green areas can be observed in both MSC-free scaffolds. This could be explained by the possible formation of a fibrotic capsule of murine origin around the scaffold implanted in the mouse, rich in collagen fibres, and/or by a possible initial osteoinductive process of the scaffolds themselves.

### 3.5 Quantifying the rate of bone formation

**Fig. 5:**
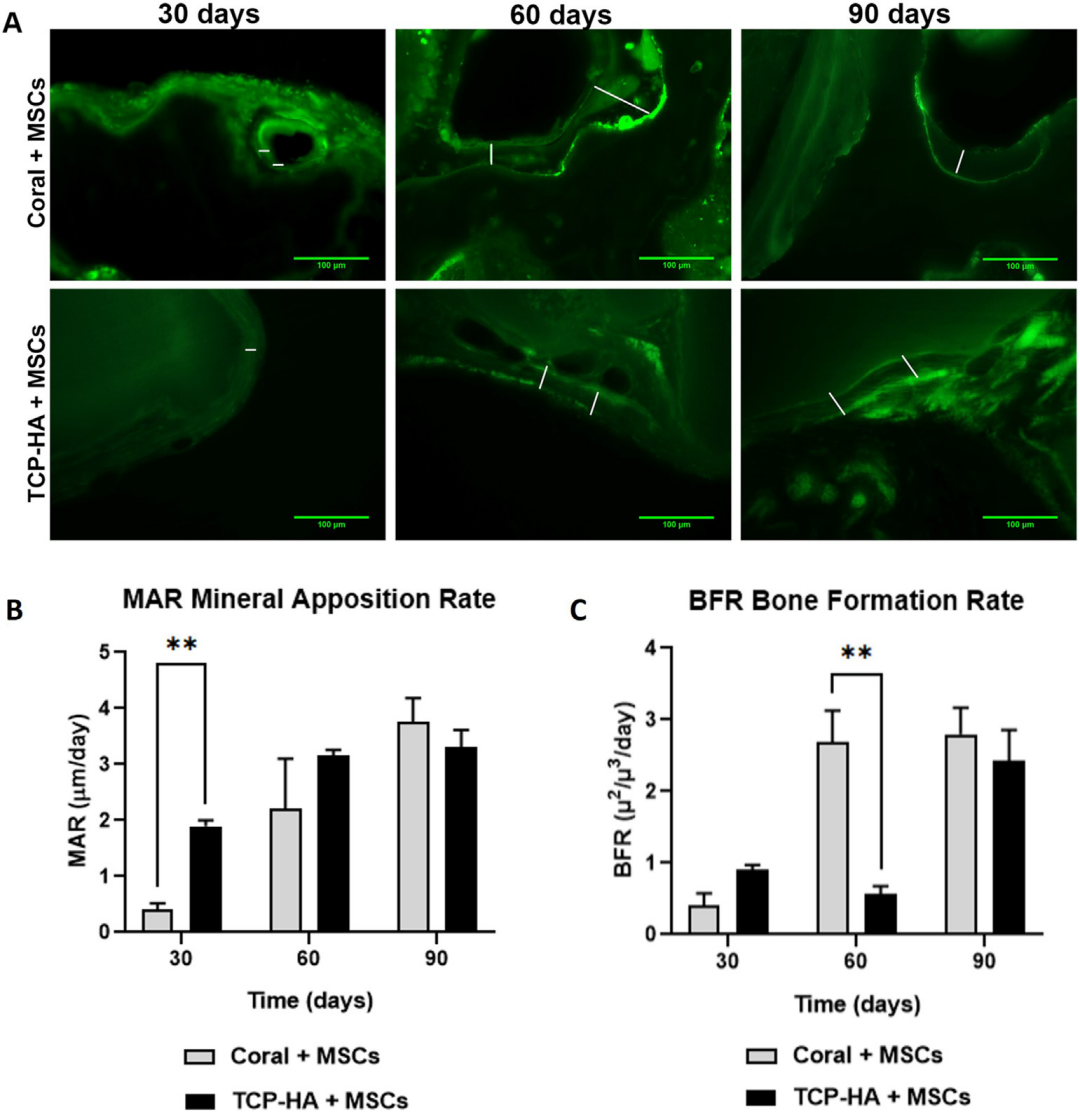
**Calcein staining in non-decalcified scaffolds.** (A) After 30, 60 and 90 days, MSC-containing coral and TCP scaffolds were sectioned and fluorescently visualized, enabling calculation of the MAR and BRF. Scale bars represent 100 μm. (B) The MAR indicated a more rapid deposition of mineral in TCP scaffolds at 30 days, then comparable MAR in both scaffold types at 60 and 90 days after implantation. (∗∗) p < 0.01, two-way ANOVA. (C) The BRF demonstrated an enhanced rate of bone formation in coral-containing explants at 60 days, an effect that normalized between both scaffold types at 90 days after implantation. (∗∗) p < 0.01, two-way ANOVA.

To assess the rate of bone formation, the mice received two intra-peritoneal injections of calcein 14 and 3 days before sacrifice. Samples explants were prepared for resin-embedding and histological staining, allowing for the observation of bone deposition dynamics in non-decalcified samples. Mineral apposition rate (MAR, μm/day) and bone formation rate were calculated (Fig. 5). MAR is the rate at which osteoblasts are depositing matrix. MAR analysis showed a statistically significant increase in rate of bone deposition in TCP-HA scaffold at 30 days, further increasing at 60 days before stabilizing at 90 days. Coral scaffolds demonstrated a steadily increasing MAR, resulting in a comparable MAR to TCP-HA at 90 days. BFR considers how much of the bone surface is actively mineralizing, which depends on the number of active osteoblasts. Interestingly, coral scaffold exhibited a statistically significant increase in BFR in scaffolds explanted after 60 days as compared to TCP-HA. These data could explain the presence of newly deposed matrix observed at late time points and reported above.

### 3.6 *In vitro* evaluation of angiogenesis

**Fig. 6:**
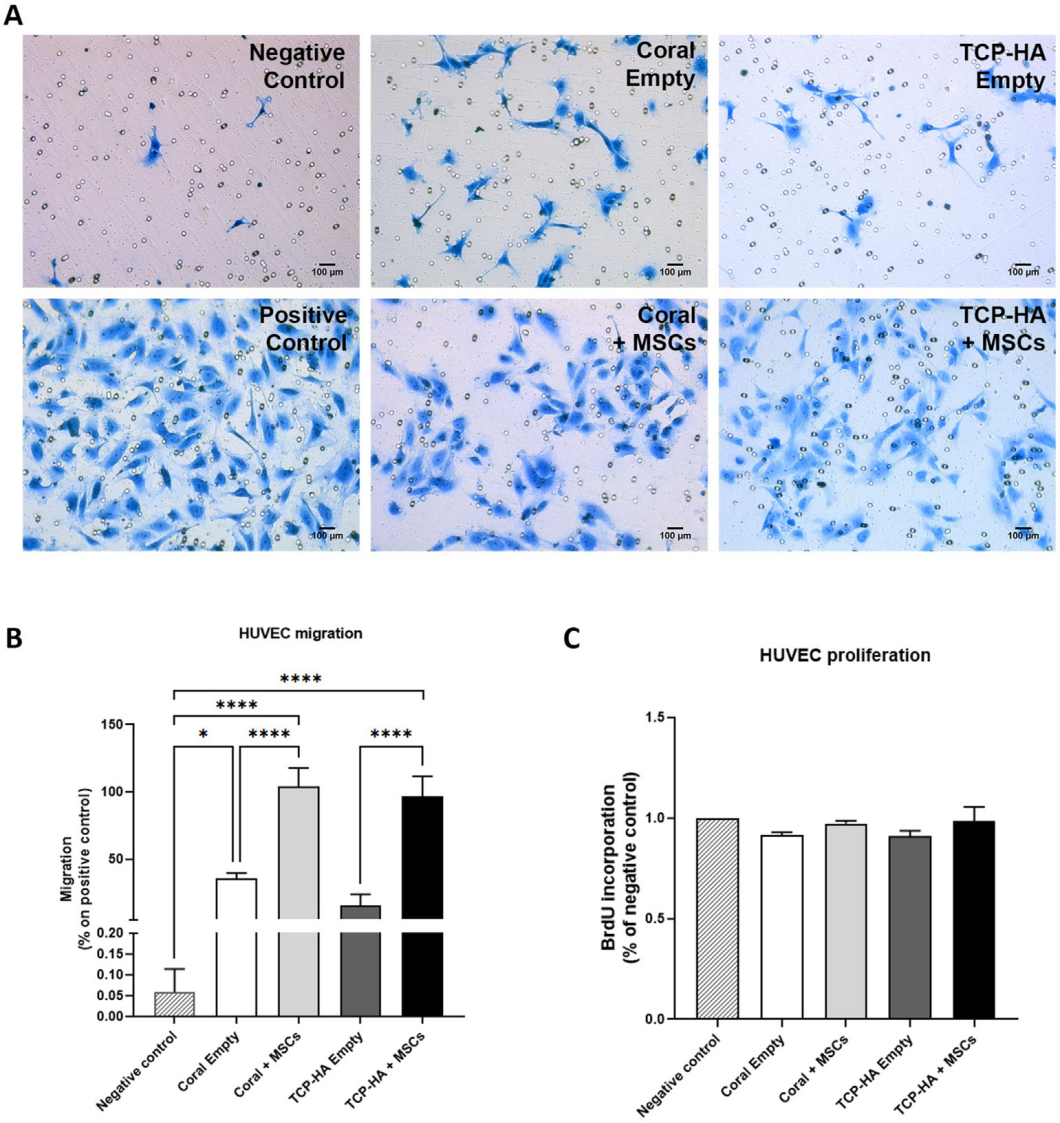
**In vitro assessment of HUVEC migration toward coral and TCP scaffolds with or without MSCs.** (A) The migration of HUVECS toward empty scaffolds (top) or MSC-seeded scaffolds (bottom) was by staining the underside of a transwell membrane. Scale bars represent 100 μm. (B) A greater number of HUVECs migrated to MSC-containing scaffolds as compared to cell-free scaffolds (C) while HUVEC proliferation rates remained constant regardless of the co-culture conditions. (∗) p < 0.05, (∗∗∗∗) p < 0.0001, one-way ANOVA.

Angiogenesis is a fundamental process for an effective bone regeneration process. Physiologically, angiogenesis starts with the budding of new capillaries from pre-existing vessels in the bone tissue. Angiogenesis is mediated by specific molecular signals such as growth factors or proteins released into the microenvironment. Therefore, coral and TCP-HA scaffolds were evaluated for their ability to induce migration and proliferation of endothelial cells (Fig. 6). Interestingly, while no effects were observed on cell proliferation (Fig. 6C), both coral and TCP-HA scaffolds were able to attract the migration of HUVECs through a permeable membrane (Fig. 6A and B). Moreover, the percentage of migrated cells was significantly higher in presence of MSCs in both coral and TCP-HA scaffolds as compared to scaffolds not containing cells, indicating that MSCs on these scaffolds are stimulated to secrete pro-angiogenic factors.

### 3.7 Vessel sprouting and homing

**Fig. 7:**
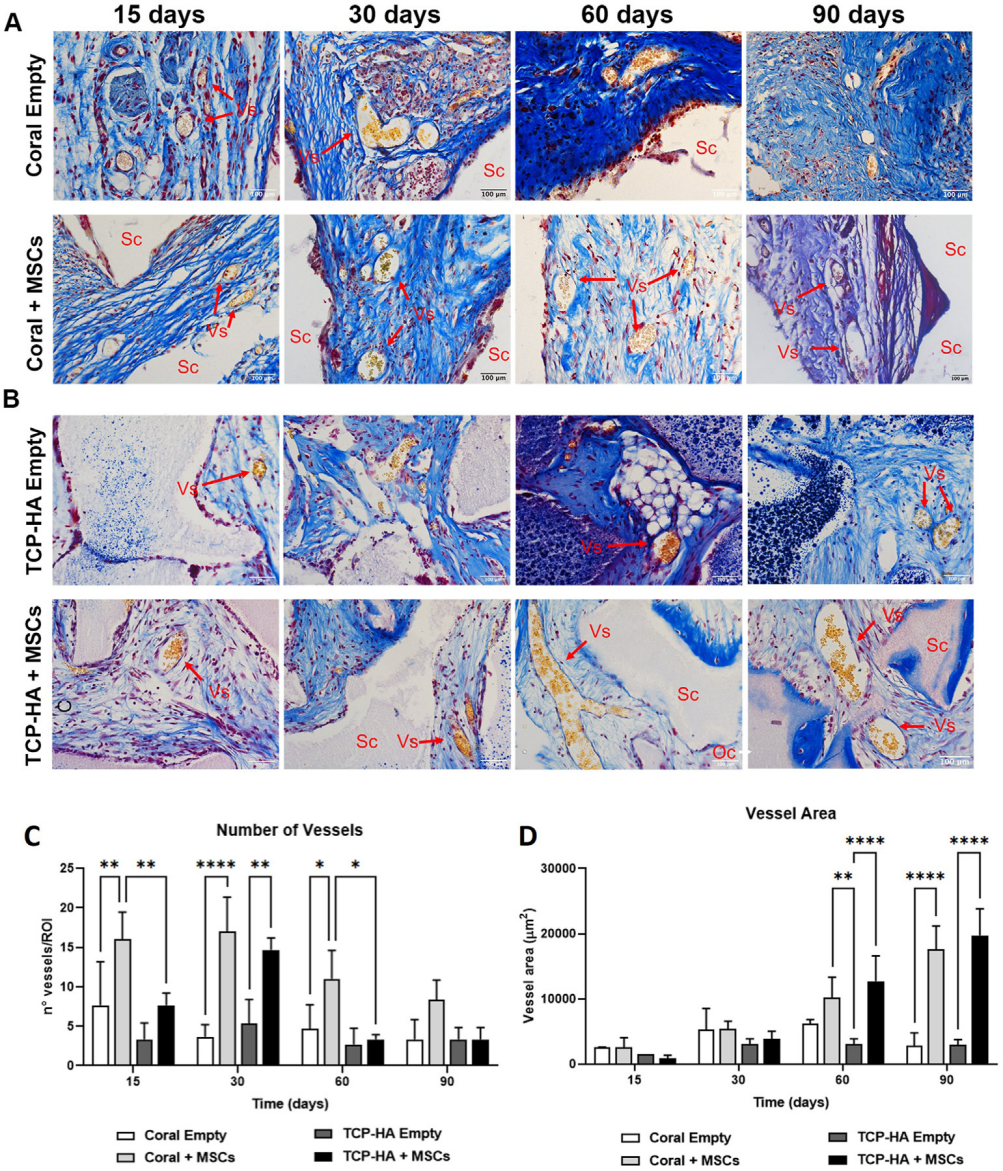
**Mallory trichrome stained explants to evaluate blood vessel number and size.** (A) Coral scaffolds with and without MSCs were compared to (B) TCP scaffolds with and without MSCs at 15, 30, 60 and 90 days *in vivo* to quantify the number and size of blood vessels (Vs) within the construct. Scale bars represent 100 μm. C) Number of blood vessels in coral and TCP scaffolds was quantified showing a decrease in number after 60 and 90 days ((∗) p < 0.05, (∗∗) p < 0.01, (∗∗∗∗) p < 0.0001, two-way ANOVA), while the vessel area (D) increased. (∗∗) p < 0.01, (∗∗∗∗) p < 0.0001, two-way ANOVA.

Detection and quantification of blood vessels sprouting inside the ceramic scaffolds were detected using Mallory trichrome staining of explanted scaffolds, followed by quantifying their number and area. At 15 days, the ingrowth of blood vessels was observed in both scaffolds (indicated by gold yellow staining), with the coral scaffold containing a statistically significant increase in the number of sprouted vessels in comparison with TCP-HA (Fig. 7A–C). Furthermore, the presence of MSCs on coral increased the number of vessels over coral scaffolds alone, demonstrating a synergistic effect. A similar number of vessels were identified after 30 days on both coral and TCP-HA scaffolds whereby the addition of MSCs resulted in a statistically significant increase in vessel number. After 60 days, the greatest number of vessels were observed in scaffolds containing coral with MSCs. After 90 days, when bone deposition has already occurred, a decrease in total number of blood vessel per area was observed in all scaffolds.

The tissue within the scaffold contained small-sized vessels after 15 and 30 days *in vivo* (Fig. 7D). With time, the vessel area increased, led by scaffolds implanted with MSCs. After 90 days, both coral and TCP-HA scaffolds with MSCs contained comparably sized vessels that were significantly larger than their acellular controls.

## 4 Discussion

Bone regeneration strategies are required to support the healing of critical bone defects as a result of trauma, infection, disease or following surgical procedures. Currently available interventions are accompanied by limitations, such as morbidity at the harvest site of autologous bone grafts, leading investigators to search for alternative bone grafting substitutes. An ideal substitute should be bioresorbable, structurally similar to trabecular bone, biocompatible, osteoconductive and osteoinductive [57]. Devitalized coral scaffolds provide a plethora of opportunities to create a bespoke bone graft substitute by tailoring the optimal combination of coral species with cellular compatibility, resorption profile and mechanical strength with its anticipated clinical application. To broaden the selection of scaffolds available for orthopaedic repair, this study investigated the biocompatibility and osteogenic potential of a sustainably grown *Pocillopora*-derived scaffold combined with adult human bone marrow derived MSCs.

The topography of *Pocillopora* scaffolds were characterized by SEM because a scaffold's texture instructs cell attachment, osteointegration and biomineralization [58]. It presented with a highly textured, granular surface containing an abundance of calice macro-pores and spinules or verrucae dispersed across the coenosteum (Fig. 1A, Supplemental Figure). The highly textured topography of these natural scaffolds is of clinical importance following reports of a positive correlation between surface roughness promoting cellular attachment [59] and osteogenic differentiation [60]. Albeit with a different cell type, topography scale and substrate, studies have shown that a landscape with protrusions comparable to *Pocillopora* verrucae inform the shape and therefore function of the cell [58]. Further, the spacing between these structures, such as the narrow grooves on *Pocillopora*, could influence osteogenic differentiation [61].

The elemental and chemical composition of *Pocillopora* scaffolds were profiled through FTIR analysis (Fig. 1B) because the elemental composition of a coral scaffold is foundational to the scaffold quality and degradation profile. The *Pocilloporidae* family of corals, including *Pocillopora,* are known for their characteristic stony appearance which is attributed to their high calcium carbonate content. In this study, the *Pocillopora* scaffold was demonstrated to be composed primarily of the aragonite form of calcium carbonate, complimenting other reports characterizing the composition of scaffolds derived from a variety of coral families [21,26]. Because calcium carbonate-based scaffolds are more rapidly absorbed *in vitro* than those composed of hydroxyapatite (HA) [62], some groups have investigated its conversion to coralline HA in order to slow its biodegradation [63,64]. Although marine scaffolds converted to HA have demonstrated biocompatibility, bioactivity and osteointegration [65], HA is poorly resorbed and will remain at the implantation site for years after it has been implanted inhibiting bone repair [24,66]. As this is the first study to characterize a *Pocillopora* scaffold *in vivo*, we have uniquely demonstrated its persistence *in vivo* in an ectopic location 8 weeks following implantation, as discussed in more detail below. Its release of calcium content during degradation may favour osteoblast proliferation and differentiation as well as extracellular matrix mineralization [67].

Mechanical analysis of large size *Pocillopora* scaffolds indicated a predictable strength distribution, related to the pore size of the samples, with a Weibull's modulus value exceeding those demonstrated in previous studies [56], indicating a more reliable failure compared to previous studies. Fracture toughness values indicate, as expected a brittle ceramic material, however, the values of fracture toughness obtained exceed those predicted for trabecular bone by over 500% [68]. This means that samples of *Pocillopora* scaffold with a similar bone architecture and pore size are considerably less likely to fracture than those of surrounding trabecular bone. This is of considerable significance, particularly in the intermediate healing scenario, where an incompletely resorbed bone graft, surrounded by newly formed trabecular bone can act as a defect if of lower fracture toughness than the newly formed bone. Compressive strengths measured of trabecular bone vary with anatomical location from 2.02 ± 0.92 MPa for vertebral locations to 17.45 ± 6.15 for femoral neck locations [69]. Compression strengths of the *Pocillopora* scaffold (21.95 ± 9.41 MPa) were comparable to compressive strengths for the strongest trabecular bone in the femoral head. Tensile strengths measured of trabecular bone vary with anatomical location from 1.72 ± 0.64 MPa for vertebral locations to 10.93 ± 3.08 for femoral neck locations [69]. Though not directly comparable, as a result of varying test methods, diametral tensile strengths of the *Pocillopora* scaffold (8.02 ± 3.60 MPa) were comparable to tensile strengths for the strongest trabecular bone in the femoral head. It should be noted that these strength values are for large sample dimensions and strengths would be expected to increase with reduced sample and maximum pore size in a predictable manner, as indicated in Fig. 2D, providing an additional factor of safety.

The biocompatibility of *Pocillopora* scaffolds was assessed by co-culturing the scaffold with adult human bone marrow derived MSCs *in vitro*, followed by quantification of cellular proliferation, metabolic activity and necrosis (Fig. 3). Adult human bone marrow derived MSCs are of specific interest to those investigating cellular therapy or tissue engineered strategies to support bone repair [70,71] and they are a cell type that orchestrates natural bone repair *in situ* [72]. The biocompatibility of other coral scaffolds with MSCs has been well characterized using *Porites* [19,27,28] and *Acropora* [24,30] where these scaffolds are understood to support MSC attachment, spread, proliferation and differentiation [39,73]. In this investigation, MSCs co-cultured with *Pocillopora* scaffolds remained viable, exhibited a fibroblastic shape and continued proliferating. It was of interest to note that exposure of MSCs to the coral scaffold for 3 days resulted in a lower cell number as compared to the untreated control, as evidenced by both live/dead staining by the reduced confluence and through quantifying DNA content. It logically flowed, then, that there was a simultaneous drop in cellular metabolism. However, there was no evidence of dying cells in the viability staining, when necrosis was quantified by LDH assays or when visualizing cells growing on the scaffolds. The reduced proliferation and metabolic activity could be the result of the initiation of osteogenic differentiation [74]. Alternatively, the lower cell number may be a technical artefact such that A) the negatively charged DNA was sequestered by the positively charged calcium carbonate, thereby depleting its availability for quantification from the lysed samples or B) that MSCs cultured adjacent to the coral scaffold integrate, thereby making the retrieval and quantification of their DNA or metabolic activity technically challenging [24,75].

Cellular adhesion is essential for the survival of cells, affecting their migration, differentiation and proliferation [76]. Surface composition, roughness, and topography also contribute to the osteogenic process, being determinants in cell contact, growth and differentiation [77,78]. Here the interaction of MSCs with the *Pocillopora* scaffolds was characterized via SEM and confocal microscopy (Fig. 3). As with coral scaffolds created from other coral species, the *Pocillopora* scaffolds investigated in this study supported cellular attachment [14,29]. By comparing SEM images of both coral scaffolds with and without the addition of MSCs (Fig. 1, Fig. 3) it was observed that MSCs thickly adhered to the granular surface of the scaffold, covering the majority of the coenosteum, demonstrating the hospitality and biocompatibility of the scaffold. The cells avoided binding to the protruding structures. While growing into the concave calices, the MSCs coordinated to physically support one another, creating a suspended membrane across the top of the external calice macropore while at the same time lining the pore (Supplemental videos). This phenotype complimented that reported by Al-Salihi et al., and Puvaneswary et al., where MSCs grew into the pores of discs created from *Porites* [41,73].

Fluorescent confocal microscopy was performed to visualize the depth and organization of the cell layers observed on the coral surface (Fig. 3E–Supplemental Video 1). Complementing the SEM imagery, an abundance of MSCs, indicated by the blue-stained nuclei, were observed on the scaffold surface. The cells were fibroblastic in shape with extending pseudopodia, indicating they have migratory capacity [79]. The MSCs were growing in layers, suggesting they were adhering both to one another as well as to the scaffold surface and the organized directionality of their phalloidin-stained cytoskeleton indicated inter-cellular communication and organization. There were also areas of scaffold where the MSCs selectively grew around the protrusions of the coral surface. While in this study, the cells organized naturally into these sheet-like structures, it is of interest to note that other investigators are engineering grafts comprised of cell sheets and coral particles, resulting in the *in vitro* formation of bone with a similar radiographic density to that of natural bone [80].

*In vivo* studies were conducted to investigate whether *Pocillopora* scaffolds supported ectopic bone formation by human bone marrow derived MSCs. Thirty days after implantation, the MSC-containing coral biomaterial contained a greater number of cells aligned in parallel to the scaffold as compared to the control MSC-seeded TCP seeded scaffolds (Fig. 4, and Supplemental Fig. 2). It is important to note that the coral constructs, after EDTA decalcification, was completely dissolved; while in TCP constructs the biomaterial remains, and the parallel orientation of the cells to the ceramic surface can be easily observed (Fig. 4, Figure Supplemental Fig. 2). This complete decalcification of the coral biomaterial could partially decrease bone recovery in our samples, with a possible loss of apparent bone formation in the area adjacent to the biomaterial. Furthermore, in this dense newly formed tissue, the presence of several blood vessels can be observed. It is known that, during development, vascularization precedes osteogenesis in both endochondral and intramembranous ossification processes [81,82]. In particular, in the coral implant it is possible to observe a large number of small vessels present already at 30 days, while in the advanced phase large calibre vessels run through the TCP scaffold. Supporting our previous observation of early bone deposition at 30 days in coral scaffolds, while at later stages at 60 and 90 days, both biomaterials showed similar vascular perfusion and bone formation (Fig. 4).

*In vivo* bone formation and remodelling can be assessed by labelling with calcium-binding calcein. These histomorphometry studies represent the gold standard method for the assessment of bone remodelling quantifying deposited bone structures. Calcein-fluorochrome, a calcium chelator that can be incorporated into the region of new bone deposition and mineralization, was detected in sections of non-decalcified specimens [83]. The presence of the calcein green label indicates the site, time, and amount of bone deposition so that the MAR and BFR can be calculated [84]. MAR analysis showed a statistically significant increase in bone deposition rate in the TCP-HA scaffold starting at 30 days, further increasing at 60 days before stabilizing at 90 days. Coral scaffolds showed similar trend, resulting in a MAR comparable to TCP-HA at 90 days. Interestingly, coral scaffold exhibited a statistically significantly higher BFR in scaffolds explanted after 60 days as compared to TCP-HA. These data could explain the presence of newly deposited bone matrix observed at subsequent time points and already described above; confirming, once again, that a peak of bone mineralization is observed between the 30th and 60th day and then became uniform with the TCP control in the late period.

The origin of the newly formed bone remains to be clarified, whether implanted MSCs or host cells contribute to tissue formation. This team has evaluated several biomaterial formulations (hydroxyapatite, TCP-HA, collagen) [85,86] in combination with human or murine MSCs [87,88] or amniotic cells [89] to study neo-bone formation. From these studies, it was discovered that the implanted human MSC can induce bone formation [51] in TCP-HA biomaterial. The internal neo-bone in close contact with the scaffold contained human MSCs, identified by the human Alu sequence. The external neo-bone was colonized by host murine cells negative for human Alu sequence). Our observation suggested that the implanted human MSC attached to the ceramics granules in the inner part of the scaffold and were responsible for initial neo-bone formation, but at the same time the human cells released factors and molecules capable of creating a microenvironment rich of host progenitor that simultaneously contributed to neo-bone formation.

Angiogenesis is a fundamental process for effective bone regeneration. Physiologically, angiogenesis starts with the budding of new capillaries from pre-existing vessels in the bone tissue [90]. Furthermore, angiogenesis is mediated by specific molecular signals such as for example growth factors or proteins released into the microenvironment [91]. In this study, both the biomaterials associated with MSC could produce factors that stimulate *in vitro* neo angiogenesis with migration and proliferation of endothelial cells (Fig. 6). As already reported in our previous manuscript that the absence of MSC results in a decrease in HUVEC number, this observation was detected in both scaffolds [51]. Given these findings, we suggested that the stimulated osteogenesis of MSCs could be triggered by calcium ions released from both scaffolds [92,93]. This event could support the paracrine effect of MSCs that modulates endothelial cells to form capillary tubes and to support angiogenesis and promote mature osteogenic differentiation in coral as well as TCP/HA scaffolds.

As with any study, there are limitations to the study presented herein. First, the findings support the development of a coral-based bone grafting substitute. However, the clinical efficacy of such a material requires verification in large animal and human clinical studies. Second, the SEM and confocal images presented demonstrate abundant cell adhesion and coverage of the scaffold material. Those cultures were performed on pieces of coral that were larger than the average 1–2 mm bone grafting substitute granule. A smaller granule has a different ratio of internal and external surface area. Our data supports the hypothesis that MSCs would adhere as readily to a smaller scaffold. Third, there is significant genetic and phenotypic variability between individual coral colonies in the wild [94] and therefore the findings for these captive specimens grown under controlled conditions may not apply to other examples of corals of the same species, or the specimens grown under different conditions. Zoan Biomed's corals are propagated clonally and grown under known and tightly controlled conditions, so future specimens of the same lineage of coral from their production system should be expected to have the same properties. Fourth, the mechanical analysis of the coral was conducted using large sample dimensions to comply with international standards. While this is good practice, the results may vary for smaller particulate samples of the same coral due to variances in pore distribution and fracture probability.

To conclude, the sustainably grown, *Pocillopora* scaffolds ware composed primarily of calcium carbonate, have a strength comparable to that of bone and are biocompatible. Most critically, coral scaffolds promoted bone formation and angiogenesis *in vivo*. Therefore, *Pocillopora* scaffolds are suitable candidates for bone graft substitutes or expanders and warrant continued investigation into its *in vivo* efficacy.

## Funding

The *in vitro* work was partially funded by Enterprise Ireland Innovation Voucher IV20163, partially by Zoan BioMed and through internal funding mechanisms. Enterprise Ireland did not have a role in study design, data collection/analysis/interpretation, writing of the report or the decision to submit this work for publication. Zoan BioMed supplied scaffold materials, purchased animals for the *in vivo* work and had an active role in writing the manuscript. KT funded by 10.13039/100014989CZI grant DAF2021-225429 and grant DOI https://doi.org/10.37921/723688hijigu from the Chan Zuckerberg Initiative DAF.

## Data availability

The raw/processed data required to reproduce these findings cannot be shared at this time due to legal or ethical reasons.

## Author contributions

CG: Data curation, Formal analysis, Funding acquisition, Investigation, Methodology, Project administration, Resources, Supervisor, Visualization, Writing – Original Draft, Reviewing and Editing.

MEFP: Data curation, Formal analysis, Investigation, Methodology, Validation, Visualization, Writing – Original Draft, Reviewing and Editing.

GS: Data curation, Formal analysis, Investigation, Methodology, Validation, Writing- Original Draft, Reviewing and Editing.

SM: Data curation, Formal analysis, Investigation, Methodology, Validation, Writing- Original Draft, Reviewing and Editing.

MS: Formal analysis, Validation, Writing- Original Draft, Reviewing and Editing.

YYO-K: Data curation, Formal analysis, Investigation, Writing- Original Draft, Reviewing and Editing.

JM: Methodology, Resources, Validation, Writing – Original Draft, Writing- Reviewing and Editing.

MJ: Methodology, Resources, Validation, Visualization, Writing – Original Draft, Writing- Reviewing and Editing.

KT: Data curation, Investigation, Methodology, Resources, Supervision, Visualization, Writing- Reviewing and Editing.

OC: Formal analysis, Investigation, Project administration, Resources, Supervision, Writing- Original Draft, Reviewing and Editing.

CMC: Conceptualization, Formal analysis, Funding acquisition, Investigation, Project administration, Resources, Supervision, Writing- Original Draft, Reviewing and Editing.

## Declaration of competing interest

The authors declare the following financial interests/personal relationships which may be considered as potential competing interests:

The majority of authors declare that they have no known competing financial interests or personal relationships that could have appeared to influence the work reported in this paper. However, two authors declare the following financial interests/personal relationships which may be considered as potential competing interests.

James Martin:

• Was employed as Production Director by Zoan BioMed during the time of this study, but no longer holds that position.
• Is inventor on 4 patents relating to the coral biomaterials used in this study, but has no direct or indirect personal gain from the impact of this study on patent values or outcomes.

Martin Johnson:

• Is the Head of R&D/CSO at Zoan BioMed, but has no direct personal gain from the impact of this work on the company.
• Is inventor on 3 patents relating to the coral biomaterials used in this study, but has no direct or indirect personal gain from the impact of this study on patent values or outcomes.
• Was co-PI with Cynthia Coleman on an Enterprise Ireland Innovation Partnership, that expired in Feb 2024.

Cynthia Coleman:

• Was co-PI with Martin Johnson on an Enterprise Ireland Innovation Partnership, that expired in Feb 2024.

## References

[1] Marsell R., Einhorn T.A. (2011). The biology of fracture healing. Injury.

[2] Avedian R.S., Haydon R.C., Peabody T.D. (2010). Multiplanar osteotomy with limited wide margins: a tissue preserving surgical technique for high-grade bone sarcomas. Clin Orthop Relat Res.

[3] Jiao H., Xiao E., Graves D.T. (2015). Diabetes and its effect on bone and fracture healing. Curr Osteoporos Rep.

[4] Ghassemi T., Shahroodi A., Ebrahimzadeh M.H., Mousavian A., Movaffagh J., Moradi A. (2018). Current concepts in scaffolding for bone tissue engineering. Arch Bone Jt Surg.

[5] Stryker (2021). Vitoss synthetic bone graft. https://www.stryker.com/us/en/spine/products/vitoss.html.

[6] James R., Deng M., Laurencin C.T., Kumbar S.G. (2011). Nanocomposites and bone regeneration. Front Mater Sci.

[7] Oryan A., Alidadi S., Moshiri A., Maffulli N. (2014). Bone regenerative medicine: classic options, novel strategies, and future directions. J Orthop Surg Res.

[8] Baldwin P., Li D.J., Auston D.A., Mir H.S., Yoon R.S., Koval K.J. (2019). Autograft, allograft, and bone graft substitutes: clinical evidence and indications for use in the setting of orthopaedic trauma surgery. J Orthop Trauma.

[9] Lobb D.C., DeGeorge B.R., Chhabra A.B. (2019). Bone graft substitutes: current concepts and future expectations. J Hand Surg Am.

[10] Sohn H.S., Oh J.K. (2019). Review of bone graft and bone substitutes with an emphasis on fracture surgeries. Biomater Res.

[11] Younger E.M., Chapman M.W. (1989). Morbidity at bone graft donor sites. J Orthop Trauma.

[12] Demers C., Hamdy C.R., Corsi K., Chellat F., Tabrizian M., Yahia L. (2002). Natural coral exoskeleton as a bone graft substitute: a review. Bio Med Mater Eng.

[13] Guillemin G., Patat J.L., Fournie J., Chetail M. (1987). The use of coral as a bone graft substitute. J Biomed Mater Res.

[14] Pountos I., Giannoudis P.V. (2016). Is there a role of coral bone substitutes in bone repair?. Injury.

[15] Green D.W., Ben-Nissan B., Yoon K.S., Milthorpe B., Jung H.S. (2017). Natural and synthetic coral biomineralization for human bone revitalization. Trends Biotechnol.

[16] Wu Y.C., Lee T.M., Chiu K.H., Shaw S.Y., Yang C.Y. (2009). A comparative study of the physical and mechanical properties of three natural corals based on the criteria for bone-tissue engineering scaffolds. J Mater Sci Mater Med.

[17] Foo L.H., Suzina A.H., Azlina A., Kannan T.P. (2008). Gene expression analysis of osteoblasts seeded in coral scaffold. J Biomed Mater Res A.

[18] Gross-Aviv T., Vago R. (2009). The role of aragonite matrix surface chemistry on the chondrogenic differentiation of mesenchymal stem cells. Biomaterials.

[19] Tran C.T., Gargiulo C., Thao H.D., Tuan H.M., Filgueira L., Michael Strong D. (2011). Culture and differentiation of osteoblasts on coral scaffold from human bone marrow mesenchymal stem cells. Cell Tissue Bank.

[20] Souyris F., Pellequer C., Payrot C., Servera C. (1985). Coral, a new biomedical material. Experimental and first clinical investigations on Madreporaria. J Maxillofac Surg.

[21] Popov A.A., Sergeeva N.S., Britaev T.A., Komlev V.S., Sviridova I.K., Kirsanova V.A. (2015). Some physical, chemical, and biological parameters of samples of scleractinium coral aquaculture skeleton used for reconstruction/engineering of the bone tissue. Bull Exp Biol Med.

[22] Sergeeva N.S., Britaev T.A., Sviridova I.K., Akhmedova S.A., Kirsanova V.A., Popov A.A. (2014). Scleractinium coral aquaculture skeleton: a possible 3D scaffold for cell cultures and bone tissue engineering. Bull Exp Biol Med.

[23] Ikeda H., Okamura T., Nishikawa T., Kobayashi N., Hashimoto Y., Tominaga K. (2023). Bone augmentation with a prototype coral exoskeleton-derived bone replacement material applied to experimental one-wall infrabony defects created in alveolar bone. Dent Mater J.

[24] Manassero M., Viateau V., Deschepper M., Oudina K., Logeart-Avramoglou D., Petite H. (2013). Bone regeneration in sheep using acropora coral, a natural resorbable scaffold, and autologous mesenchymal stem cells. Tissue Eng Part A.

[25] Ramdan R., Sunendar B., Hermawan H. (2016).

[26] Guillemin G., Meunier A., Dallant P., Christel P., Pouliquen J.C., Sedel L. (1989). Comparison of coral resorption and bone apposition with two natural corals of different porosities. J Biomed Mater Res.

[27] Bensaid W., Oudina K., Viateau V., Potier E., Bousson V., Blanchat C. (2005). De novo reconstruction of functional bone by tissue engineering in the metatarsal sheep model. Tissue Eng.

[28] Petite H., Viateau V., Bensaid W., Meunier A., de Pollak C., Bourguignon M. (2000). Tissue-engineered bone regeneration. Nat Biotechnol.

[29] Viateau V., Guillemin G., Bousson V., Oudina K., Hannouche D., Sedel L. (2007). Long-bone critical-size defects treated with tissue-engineered grafts: a study on sheep. J Orthop Res.

[30] Decambron A., Manassero M., Bensidhoum M., Lecuelle B., Logeart-Avramoglou D., Petite H. (2017). A comparative study of tissue-engineered constructs from Acropora and Porites coral in a large animal bone defect model. Bone Joint Res.

[31] Vuola J., Bohling T., Kinnunen J., Hirvensalo E., Asko-Seljavaara S. (2000). Natural coral as bone-defect-filling material. J Biomed Mater Res.

[32] Zhang S., Mao T., Chen F. (2011). Influence of platelet-rich plasma on ectopic bone formation of bone marrow stromal cells in porous coral. Int J Oral Maxillofac Surg.

[33] Tawfeek G.A., Abdelgaber M., Gadallah S., Anis A., Sharshar A. (2023). A novel construct of coral granules-poly-L-lactic acid nanomembrane sandwich double stem cell sheet transplantation as regenerative therapy of bone defect model. Exp Clin Transplant.

[34] Decambron A., Fournet A., Bensidhoum M., Manassero M., Sailhan F., Petite H. (2017). Low-dose BMP-2 and MSC dual delivery onto coral scaffold for critical-size bone defect regeneration in sheep. J Orthop Res.

[35] Hou R., Chen F., Yang Y., Cheng X., Gao Z., Yang H.O. (2007). Comparative study between coral-mesenchymal stem cells-rhBMP-2 composite and auto-bone-graft in rabbit critical-sized cranial defect model. J Biomed Mater Res A.

[36] Geiger F., Lorenz H., Xu W., Szalay K., Kasten P., Claes L. (2007). VEGF producing bone marrow stromal cells (BMSC) enhance vascularization and resorption of a natural coral bone substitute. Bone.

[37] Xiao C., Zhou H., Liu G., Zhang P., Fu Y., Gu P. (2011). Bone marrow stromal cells with a combined expression of BMP-2 and VEGF-165 enhanced bone regeneration. Biomed Mater.

[38] Mohan S., Karunanithi P., Raman Murali M., Anwar Ayob K., Megala J., Genasan K. (2022). Potential use of 3D CORAGRAF-loaded PDGF-BB in PLGA microsphere seeded mesenchymal stromal cells in enhancing the repair of calvaria critical-size bone defect in rat model. Mar Drugs.

[39] Sautier J.M., Nefussi J.R., Boulekbache H., Forest N. (1990). In vitro bone formation on coral granules. In Vitro Cell Dev Biol.

[40] Cui L., Liu B., Liu G., Zhang W., Cen L., Sun J. (2007). Repair of cranial bone defects with adipose derived stem cells and coral scaffold in a canine model. Biomaterials.

[41] Puvaneswary S., Balaji Raghavendran H.R., Ibrahim N.S., Murali M.R., Merican A.M., Kamarul T. (2013). A comparative study on morphochemical properties and osteogenic cell differentiation within bone graft and coral graft culture systems. Int J Med Sci.

[42] Louisia S., Stromboni M., Meunier A., Sedel L., Petite H. (1999). Coral grafting supplemented with bone marrow. J Bone Joint Surg Br.

[43] Viateau V., Manassero M., Sensebe L., Langonne A., Marchat D., Logeart-Avramoglou D. (2016). Comparative study of the osteogenic ability of four different ceramic constructs in an ectopic large animal model. J Tissue Eng Regen Med.

[44] Martin J., Office E.P. (2021). Bone graft substitutes.

[45] Chow L.C., Hirayama S., Takagi S., Parry E. (2000). Diametral tensile strength and compressive strength of a calcium phosphate cement: effect of applied pressure. J Biomed Mater Res.

[46] Wang L., D'Alpino P.H., Lopes L.G., Pereira J.C. (2003). Mechanical properties of dental restorative materials: relative contribution of laboratory tests. J Appl Oral Sci.

[47] Taylor D. (2007).

[48] Taylor D., Walsh M., Cullen A., O'Reilly P. (2016). The fracture toughness of eggshell. Acta Biomater.

[49] Cassidy F.C., Shortiss C., Murphy C.G., Kearns S.R., Curtin W., De Buitleir C. (2020). Impact of type 2 diabetes mellitus on human bone marrow stromal cell number and phenotypic characteristics. Int J Mol Sci.

[50] Creane M., Howard L., O'Brien T., Coleman C.M. (2017). Biodistribution and retention of locally administered human mesenchymal stromal cells: quantitative polymerase chain reaction-based detection of human DNA in murine organs. Cytotherapy.

[51] Pereira R.C., Benelli R., Canciani B., Scaranari M., Daculsi G., Cancedda R. (2019). Beta-tricalcium phosphate ceramic triggers fast and robust bone formation by human mesenchymal stem cells. J Tissue Eng Regen Med.

[52] Stolarski J., Coronado I., Murphy J.G., Kitahara M.V., Janiszewska K., Mazur M. (2021). A modern scleractinian coral with a two-component calcite-aragonite skeleton. Proc Natl Acad Sci U S A.

[53] Von Euw S., Zhang Q., Manichev V., Murali N., Gross J., Feldman L.C. (2017). Biological control of aragonite formation in stony corals. Science.

[54] Chakrabarty D., Mahapatra S. (1999). Aragonite crystals with unconventional morphologies. J Mater Chem.

[55] Gbureck U., Hözel T., Klammert U., Würzler K., Müller F.A., Barralet J.E. (2007). Resorbable dicalcium phosphate bone substitutes prepared by 3D powder printing. Adv Funct Mater.

[56] Ma L.J., Li Z., Wang M.Y., Wei H.Z., Fan P.X. (2019). Effects of size and loading rate on the mechanical properties of single coral particles. Powder Technol.

[57] Giannoudis P.V., Dinopoulos H., Tsiridis E. (2005). Bone substitutes: an update. Injury.

[58] Barata D., Dias P., Wieringa P., van Blitterswijk C., Habibovic P. (2017). Cell-instructive high-resolution micropatterned polylactic acid surfaces. Biofabrication.

[59] Deng Y., Liu X., Xu A., Wang L., Luo Z., Zheng Y. (2015). Effect of surface roughness on osteogenesis in vitro and osseointegration in vivo of carbon fiber-reinforced polyetheretherketone-nanohydroxyapatite composite. Int J Nanomedicine.

[60] Hatano K., Inoue H., Kojo T., Matsunaga T., Tsujisawa T., Uchiyama C. (1999). Effect of surface roughness on proliferation and alkaline phosphatase expression of rat calvarial cells cultured on polystyrene. Bone.

[61] Sun L., Pereira D., Wang Q., Barata D.B., Truckenmuller R., Li Z. (2016). Controlling growth and osteogenic differentiation of osteoblasts on microgrooved polystyrene surfaces. PLoS One.

[62] Combes C., Bareille R., Rey C. (2006). Calcium carbonate-calcium phosphate mixed cement compositions for bone reconstruction. J Biomed Mater Res A.

[63] Pollick S., Shors E.C., Holmes R.E., Kraut R.A. (1995). Bone formation and implant degradation of coralline porous ceramics placed in bone and ectopic sites. J Oral Maxillofac Surg.

[64] Ripamonti U., Crooks J., Khoali L., Roden L. (2009). The induction of bone formation by coral-derived calcium carbonate/hydroxyapatite constructs. Biomaterials.

[65] Zhang X., Vecchio K.S. (2013). Conversion of natural marine skeletons as scaffolds for bone tissue engineering. Front Mater Sci.

[66] Hasegawa S., Ishii S., Tamura J., Furukawa T., Neo M., Matsusue Y. (2006). A 5-7 year in vivo study of high-strength hydroxyapatite/poly(L-lactide) composite rods for the internal fixation of bone fractures. Biomaterials.

[67] Maeno S., Niki Y., Matsumoto H., Morioka H., Yatabe T., Funayama A. (2005). The effect of calcium ion concentration on osteoblast viability, proliferation and differentiation in monolayer and 3D culture. Biomaterials.

[68] Yan L., Cinar A., Ma S., Abel R., Hansen U., Marrow T.J. (2020). A method for fracture toughness measurement in trabecular bone using computed tomography, image correlation and finite element methods. J Mech Behav Biomed Mater.

[69] Morgan E.F., Keaveny T.M. (2001). Dependence of yield strain of human trabecular bone on anatomic site. J Biomech.

[70] Lin H., Sohn J., Shen H., Langhans M.T., Tuan R.S. (2019). Bone marrow mesenchymal stem cells: aging and tissue engineering applications to enhance bone healing. Biomaterials.

[71] Wang J., Chen Z., Sun M., Xu H., Gao Y., Liu J. (2020). Characterization and therapeutic applications of mesenchymal stem cells for regenerative medicine. Tissue Cell.

[72] Watson L., Elliman S.J., Coleman C.M. (2014). From isolation to implantation: a concise review of mesenchymal stem cell therapy in bone fracture repair. Stem Cell Res Ther.

[73] Al-Salihi K.A., Samsudin A.R. (2004). Bone marrow mesenchymal stem cells differentiation and proliferation on the surface of coral implant. Med J Malaysia.

[74] Maia F.R., Lourenco A.H., Granja P.L., Goncalves R.M., Barrias C.C. (2014). Effect of cell density on mesenchymal stem cells aggregation in RGD-alginate 3D matrices under osteoinductive conditions. Macromol Biosci.

[75] Biocoral (2020). Biocoral product information sheet. https://biocoral.com/wp-content/uploads/2019/06/Universe-of-Biocoral-VAnglaise-Doc-51-5-17-General.pdf.

[76] Nguyen A.T., Sathe S.R., Yim E.K. (2016). From nano to micro: topographical scale and its impact on cell adhesion, morphology and contact guidance. J Phys Condens Matter.

[77] Dalby M.J., Andar A., Nag A., Affrossman S., Tare R., McFarlane S. (2008). Genomic expression of mesenchymal stem cells to altered nanoscale topographies. J R Soc Interface.

[78] Dalby M.J., McCloy D., Robertson M., Wilkinson C.D., Oreffo R.O. (2006). Osteoprogenitor response to defined topographies with nanoscale depths. Biomaterials.

[79] Ryan C.M., Brown J.A., Bourke E., Prendergast A.M., Kavanagh C., Liu Z. (2015). ROCK activity and the Gbetagamma complex mediate chemotactic migration of mouse bone marrow-derived stromal cells. Stem Cell Res Ther.

[80] Geng W., Ma D., Yan X., Liu L., Cui J., Xie X. (2013). Engineering tubular bone using mesenchymal stem cell sheets and coral particles. Biochem Biophys Res Commun.

[81] Kronenberg H.M. (2003). Developmental regulation of the growth plate. Nature.

[82] Zelzer E., Mamluk R., Ferrara N., Johnson R.S., Schipani E., Olsen B.R. (2004). VEGFA is necessary for chondrocyte survival during bone development. Development.

[83] Shim M.-J. (2016). Bone changes in femoral bone of mice using calcein labeling. Korean J Clinic Labor Sci.

[84] Aido M., Kerschnitzki M., Hoerth R., Burghammer M., Montero C., Checa S. (2014). Relationship between nanoscale mineral properties and calcein labeling in mineralizing bone surfaces. Connect Tissue Res.

[85] Mastrogiacomo M., Papadimitropoulos A., Cedola A., Peyrin F., Giannoni P., Pearce S.G. (2007). Engineering of bone using bone marrow stromal cells and a silicon-stabilized tricalcium phosphate bioceramic: evidence for a coupling between bone formation and scaffold resorption. Biomaterials.

[86] Muraglia A., Martin I., Cancedda R., Quarto R. (1998). A nude mouse model for human bone formation in unloaded conditions. Bone.

[87] Tasso R., Augello A., Boccardo S., Salvi S., Carida M., Postiglione F. (2009). Recruitment of a host's osteoprogenitor cells using exogenous mesenchymal stem cells seeded on porous ceramic. Tissue Eng Part A.

[88] Tortelli F., Tasso R., Loiacono F., Cancedda R. (2010). The development of tissue-engineered bone of different origin through endochondral and intramembranous ossification following the implantation of mesenchymal stem cells and osteoblasts in a murine model. Biomaterials.

[89] Mirabella T., Poggi A., Scaranari M., Mogni M., Lituania M., Baldo C. (2011). Recruitment of host's progenitor cells to sites of human amniotic fluid stem cells implantation. Biomaterials.

[90] Diomede F., Marconi G.D., Fonticoli L., Pizzicanella J., Merciaro I., Bramanti P. (2020). Functional relationship between osteogenesis and angiogenesis in tissue regeneration. Int J Mol Sci.

[91] Li J., Zhang Y.P., Kirsner R.S. (2003). Angiogenesis in wound repair: angiogenic growth factors and the extracellular matrix. Microsc Res Tech.

[92] Habraken W., Habibovic P., Epple M., Bohner M. (2016). Calcium phosphates in biomedical applications: materials for the future?. Mater Today.

[93] Klar R.M., Duarte R., Dix-Peek T., Dickens C., Ferretti C., Ripamonti U. (2013). Calcium ions and osteoclastogenesis initiate the induction of bone formation by coral-derived macroporous constructs. J Cell Mol Med.

[94] Schmidt-Roach S., Lundgren P., Miller K.J., Gerlach G., Noreen A.M.E., Andreakis N. (2012). Assessing hidden species diversity in the coral Pocillopora damicornis from Eastern Australia. Coral Reefs.

